# Carbon source competition within the wound microenvironment can significantly influence infection progression

**DOI:** 10.1038/s41522-024-00518-4

**Published:** 2024-06-25

**Authors:** Evgenia Maslova, Lara EisaianKhongi, Petra Rigole, Tom Coenye, Ronan R. McCarthy

**Affiliations:** 1 Division of Biosciences, Department of Life Sciences, College of Health and Life Sciences, Brunel University London, Uxbridge, UK; 2 Laboratory of Pharmaceutical Microbiology, Ghent University, 9000 Ghent, Belgium

**Keywords:** Bacteriology, Biofilms, Pathogens

## Abstract

It is becoming increasingly apparent that commensal skin bacteria have an important role in wound healing and infection progression. However, the precise mechanisms underpinning many of these probiotic interactions remain to be fully uncovered. In this work, we demonstrate that the common skin commensal *Cutibacterium acnes* can limit the pathogenicity of the prevalent wound pathogen *Pseudomonas aeruginosa* in vivo. We show that this impact on pathogenicity is independent of any effect on growth, but occurs through a significant downregulation of the Type Three Secretion System (T3SS), the primary toxin secretion system utilised by *P. aeruginosa* in eukaryotic infection. We also show a downregulation in glucose acquisition systems, a known regulator of the T3SS, suggesting that glucose availability in a wound can influence infection progression. *C. acnes* is well known as a glucose fermenting organism, and we demonstrate that topically supplementing a wound with glucose reverses the probiotic effects of *C. acnes*. This suggests that introducing carbon source competition within the wound microenvironment may be an effective way to prevent or limit wound infection.

## Introduction

The skin is a key barrier that provides physical protection against environmental insults and opportunistic pathogens. Damage to this barrier significantly increases the likelihood of infection, which can lead to serious acute complications such as bacteraemia and sepsis if untreated^1,2^. If treatment of the infected wound with frontline therapeutics fails, wound infections can become chronic, leading to further treatment failures and prolonged hospital stays. It has been estimated that around 1–2% of the general population in developed countries suffer from chronic wounds across all age groups^3,4^. This creates major pressure on the economy but also has a significant detrimental effect on the patient’s quality of life and wellbeing^4,5^. The most commonly isolated bacterial pathogens from wounds are *Staphylococcus aureus*, *Pseudomonas aeruginosa, Klebsiella pneumoniae, Enterobacter spp, Enterococcus faecalis* and *Acinetobacter baumannii*^6–8^. These bacteria belong to the ESKAPE pathogens, a watchlist of pathogens that are rapidly gaining antibiotic resistance. These pathogens also feature on the World Health Organisations Priority List for which there is an urgent need to develop alternative treatment strategies^9–13^.

The skin microbiome is one of the largest bacterial communities in the human body and plays an important role in the maintenance of the skin barrier and in regulating wound healing^12,14,15^. Amongst the commensal skin microbiota, several commensal species can occasionally lead to opportunistic infection including *Staphylococcus epidermidis, Streptococcus mitis, Cutibacterium (*formerly *Propionibacterium) acnes, Corynebacterium spp* and *Acinetobacter johnsonii*^16–18^. Some true pathogens are also part of the skin microbiome, including *S. aureus, P. aeruginosa* and *Streptococcus pyogenes*; however, colonisation of intact skin with these pathogens tends to be asymptomatic and transient^19,20^.

Disruption of skin integrity provides an opportunity for commensal skin bacteria and environmental microorganisms to colonise the wound bed soon after the injury^21^. Wound beds are a nutrient-rich environment that provides access to deeper tissues for microorganisms to colonise and thrive^22,23^. Early wound colonisation by commensal non-pathogenic bacteria has been described as beneficial due to the principle of competitive exclusion in which some species out-compete each other for the space and nutrients available in a wound^24^. *Lactobacillus reuteri* has been shown to inhibit *S. aureus* adherence via competitive exclusion in a keratinocyte co-culture model^25^. Additionally, vancomycin-induced skin microbiota dysbiosis in mice was reported to delay wound healing, implying that the skin microbiota is important in the process of wound healing^26^. Furthermore, wounds that displayed a dysbiotic skin microbiome were reported to exhibit increased inflammation and delayed healing in comparison to healthy microbiome wounds in rats^27^.

*C. acnes* is a commensal organism but can cause acne vulgaris and various opportunistic infections, e.g. surgical site infections^28,29^. *C. acnes* strains are categorised into several phylotypes depending on the expression of putative virulence factors, i.e., IA1 is typically associated with acneic skin, whereas IA2, IB, IC and II are associated with healthy skin^30,31^. Phylotype I has been associated with moderate-to-severe acne, whereas all other phylotypes are either isolated from soft tissue infections or normal skin microbiota and are considered true commensals^32,33^. *C. acnes* has been reported to inhibit several pathogenic bacteria via the production of secreted chemicals, such as short-chain fatty acids (SCFAs), propionic acid and bacteriocins. For example, short-chain fatty acids produced by *C. acnes* have been reported to inhibit biofilm formation and decrease the bioburden of *S. aureus* in wounds^34,35^. *C. acnes* isolates have also been shown to alter *S. aureus* antibiotic susceptibility and prevent the maturation of *S. aureus* biofilms which increased their susceptibility to antimicrobial treatments^36^. *C. acnes* produces bacteriocins that have an antibiotic effect on *S. epidermidis*^37^. Production of propionic acid by *C. acnes* has been shown to kill *S. aureus* and lower the pH of the wound^38,39^. Intriguingly, no antibacterial effects against Gram-negative bacteria have been reported. In the present study we investigate the antivirulence effects of *C. acnes* isolates on the common wound pathogen *P. aeruginosa*. We propose that this effect is achieved via carbon-source competition within the wound, thus introducing the concept of carbon-source competition within the wound as a potential alternative treatment strategy for *P. aeruginosa* wound infections.

## Results

### *C. acnes* strains decrease *P. aeruginosa* PA14 induced mortality in burn infection in *G. mellonella*

**Fig. 1:**
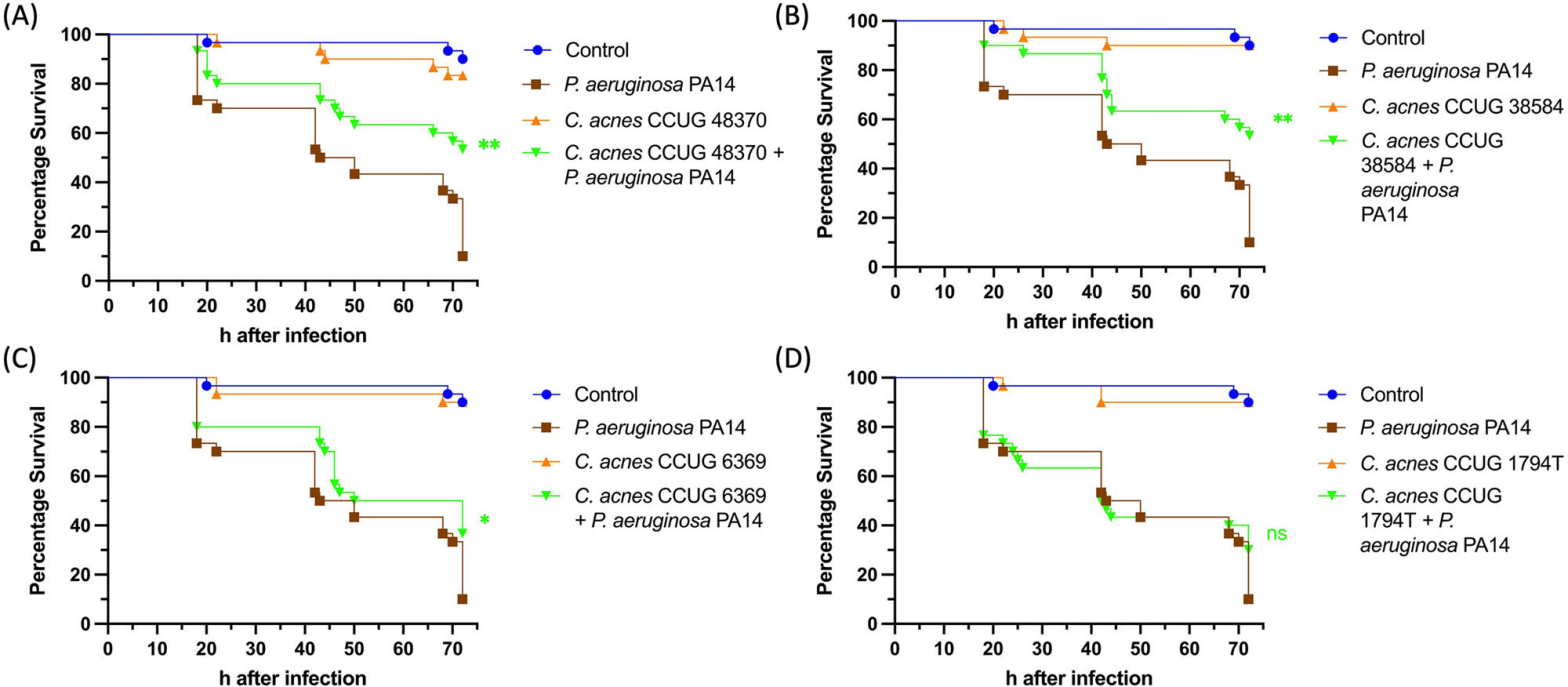
Kaplan–Meier Survival curves of in vivo *P. aeruginosa* PA14 burn infection using the *G. mellonella* burn wound infection model. **A** *P. aeruginosa* PA14 vs. *C. acnes* CCUG 48370, Log rank *p* value < 0.005. **B** *P. aeruginosa* PA14 vs. *C. acnes* CCUG 38584, Log rank *p* value < 0.005. **C** *P. aeruginosa* PA14 vs. *C. acnes* CCUG 6369, Log rank *p* value < 0.05. **D** *P. aeruginosa* PA14 vs. *C. acnes* CCUG 1794T, *p* value > 0.05. The specimens were monitored for 72 h starting at 18 h; *n* = 30 as three biological replicates of 10 larvae per experimental group. Log-rank (Mantel–Cox) test was performed to assess the statistical significance with Bonferroni correction, ns = *p* value > 0.05, **p value < 0.005.

Specific species of commensal skin bacteria, including *C. acnes* have been identified as pioneer colonisers of the wound bed that can promote healing^18,40^. To explore the capacity of *C. acnes* to limit infection progression, the invertebrate burn wound model *G. mellonella* was chosen. This model has been extensively validated as a robust tool to study burn wound infection and unlike other in vivo burn wound models it can be used to screen for novel wound therapeutics including probiotic bacteria^41–44^. A panel of *C. acnes* strains comprised of *C. acnes* CCUG 1794T, CCUG 38584, CCUG 48370, CCUG 6369 was selected to assay in this model^45^ (Supplementary Table 1). After establishing burn wounds on *G. mellonella* larvae, the wounds were seeded with each of the *C. acnes* strains. None of the *C. acnes* strains had a significant impact on larval survival confirming their status as commensals. To assess their impact on the pathogenicity of the burn wound isolate *P. aeruginosa* PA14, the *G. mellonella* burn wound assays were repeated with *P. aeruginosa* PA14 being applied to the wound immediately after *C. acnes* seeding. Wounds without *C. acnes* added but infected with *P. aeruginosa* PA14 were used as a positive control. Administration of *P. aeruginosa* PA14 alone led to 90% mortality after 72 h as expected. Larvae treated with *C. acnes* CCUG 48370 and *C. acnes* CCUG 38584 prior to infection with *P. aeruginosa* PA14 exhibited a significant 43% increase in survival, while larvae treated with *C. acnes* CCUG 6369 displayed a 20% increase in survival (Fig. 1 ABC). Larvae treated with *C. acnes* CCUG 1794T prior to infection exhibited no significant decrease in mortality, demonstrating that this reduced virulence effect was strain specific (Fig. 1 D).

### *C. acnes* decreases *P. aeruginosa* biofilm formation but does not impact growth

**Fig. 2:**
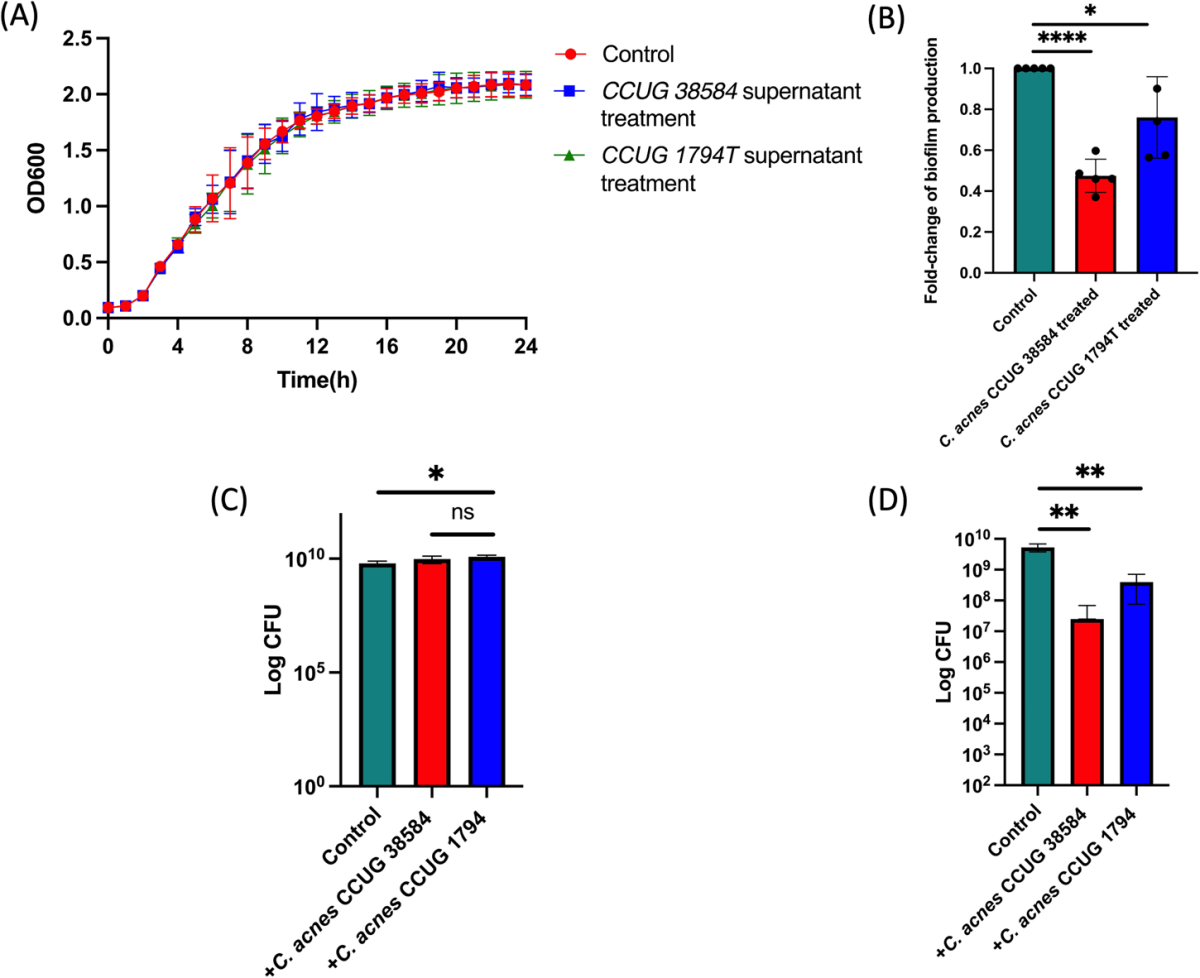
Impact of *C. acnes* on *P. aeruginosa* growth and biofilm formation. **A** Growth curve of *P. aeruginosa* PA14 liquid cultures in the presence of *C. acnes* strains supernatants (50%) showing no significant difference in the growth rates between exposures. Two-way ANOVA with multiple comparisons was performed to assess statistical significance. *P* value > 0.05 was determined as insignificant (ns). **B** The effect on biofilm formation of *P. aeruginosa* PA14 in the presence of *C. acnes* strains supernatants after 24 h. *C. acnes* CCUG 38584 supernatant treatment exhibited a 52.5% reduction of the biofilm production in *P. aeruginosa* PA14, whereas *C. acnes* CCUG 1794T exhibited a less significant 24% reduction in comparison to the control treatment. Unpaired Student’s *t* test (two tailed) was performed to assess statistical significance. ****p value < 0.0001, *p value < 0.05. Error Bars are Standard Deviation. **C** Growth of *P. aeruginosa* PA14 in an artificial sebum model in the presence of *C. acnes* CCUG 1794T and CCUG 38584 cultures. No negative effects on growth were observed. Unpaired Student’s *t* test (two tailed) was performed to assess statistical significance *p value < 0.05. Error Bars are Standard Deviation. **D** Biofilm formation of *P. aeruginosa* PA14 in an artificial sebum model in the presence of *C. acnes* CCUG 1794T and CCUG 38584 cultures after 48 h. Unpaired Student’s *t* test (two tailed) was performed to assess statistical significance **p value < 0.01. Error Bars are Standard Deviation.

*C. acnes* is known to be able to secrete a range of antibacterial compounds^18,46^. To explore if *C. acnes* was limiting *P. aeruginosa* growth and whether this was influencing infection progression, the growth of *P. aeruginosa* was monitored in the presence of the supernatant of *C. acnes* CCUG 38584, a strain that had a major impact on infection progression and *C. acnes* CCUG 1794T, a strain that did not have a significant impact on infection progression (Fig. 2A). Growth curves of *P. aeruginosa* PA14 obtained in media supplemented with 50% supernatant were not significantly different from those obtained in the absence of supernatant indicating that *C. acnes* had no impact on *P. aeruginosa* growth.

*C. acnes* has previously been shown to impact biofilm formation in Gram-positive bacteria including *S. aureus* and *S. epidermidis*; however, the specific strains tested did not affect *P. aeruginosa* PAO1 biofilm formation^34,36^. To explore the effect of *C. acnes* on biofilm formation by *P. aeruginosa* PA14, a biofilm assay was conducted in media supplemented with 50% *C. acnes* supernatant. The presence of the supernatant of *C. acnes* CCUG 38584 led to a significant reduction in biofilm formation by 52.5% in PA14 (Fig. 2B). The supernatant of *C. acnes* CCUG 1794T also significantly reduced biofilm formation, albeit to a lesser extent (24% inhibition) (Fig. 2B).

To more accurately replicate the conditions observed in a human wound and to explore if contact-dependent antagonistic mechanisms could be influencing *P. aeruginosa* growth, *P. aeruginosa* was co-cultured with *C. acnes* CCUG 38584 and *C. acnes* CCUG 1794T in an artificial sebum model (Fig. 2C). Co-culture with *C. acnes* strains had no negative effect on growth of PA14 however in agreement with our previous assays, biofilm formation was inhibited during co-culture, with *C. acnes* CCUG 38584 again having a greater effect than *C. acnes* CCUG 1794T (Fig. 2D). The lack of an inhibiting effect of *C. acnes* strains on *P. aeruginosa* PA14 growth suggests that the therapeutic effect of *C. acnes* is not due to restriction of growth of *P. aeruginosa* PA14. However, the reduction of the biofilm formation by *P. aeruginosa* PA14 in the presence of *C. acnes* could be a contributing factor to the reduction in mortality in vivo.

### *C. acnes* CCUG 38584 significantly alters the expression of important virulence and metabolism pathways of *P. aeruginosa*

**Fig. 3:**
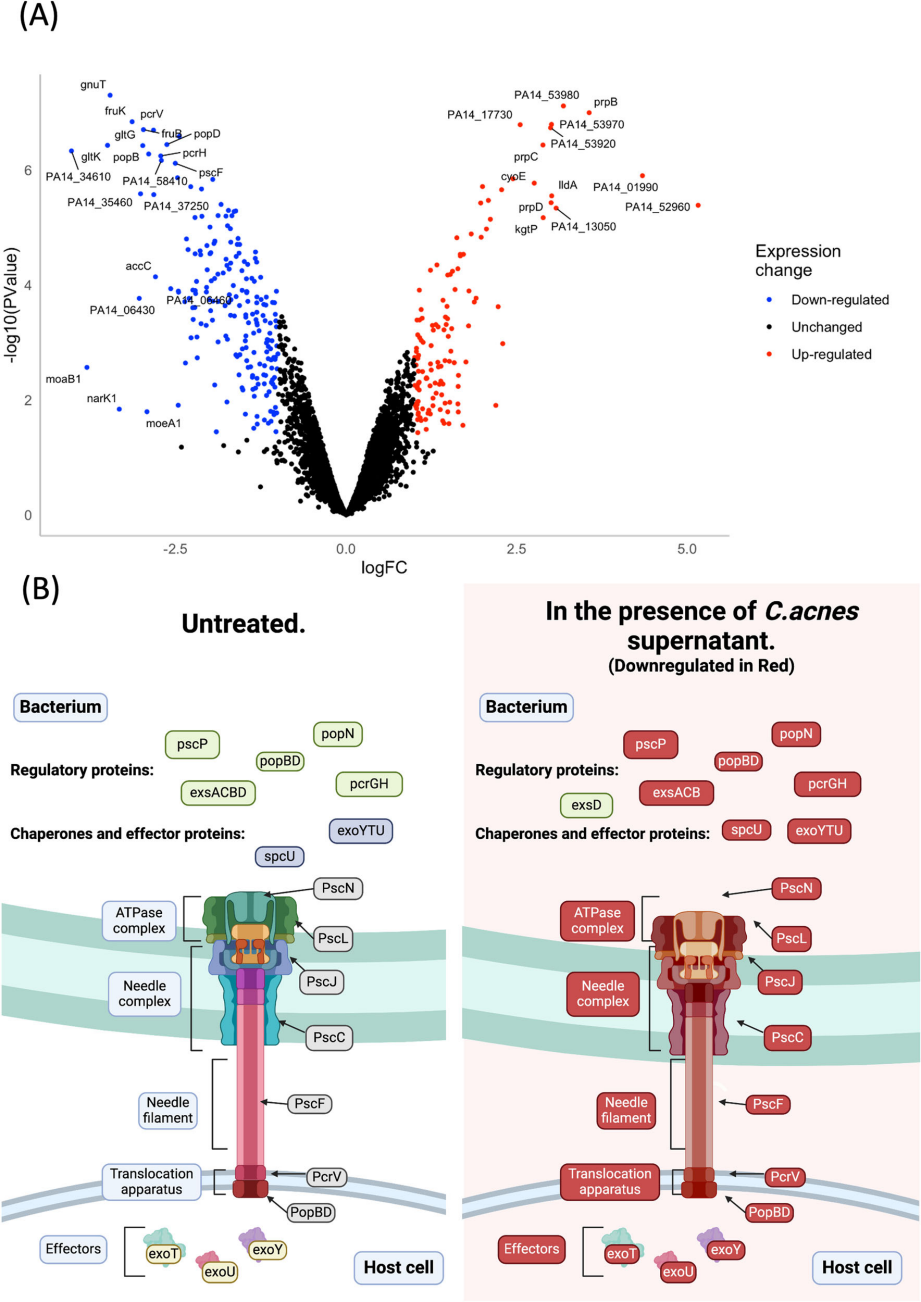
*The influence of C. acnes supernatant on P. aeruginosa gene expression.* **A** Volcano plot representing results of differential gene expression analysis of *P. aeruginosa* PA14 in the presence of *C. acnes* CCUG 38584 versus TSB control. Quantified genes were differentially expressed when Log-fold change > |1| and *p* value < 0.05. Genes which exhibited Log-fold change > |2.5| and *p* value < 0.05 are labelled. **B** A schematic representation of the T3SS in *P. aeruginosa* with the individual protein components labelled. In the presence of *C. acnes* CCUG 38584 supernatant, most of the key genes encoding for this secretion system are downregulated and the expression of this system is suppressed. Created with BioRender.com.

To explore the *P. aeruginosa* transcriptional response to exposure to *C. acnes* supernatant and potentially elucidate the cause of the antivirulence effect, RNA-seq analysis was performed on *P. aeruginosa* PA14 grown in the presence of 50% *C. acnes* CCUG 38584 supernatant. The samples were grown to mid-exponential phase before RNA was extracted and RNA-Seq performed. As a result, 334 *P. aeruginosa* genes were differentially expressed in the presence of supernatant of *C. acnes* CCUG 38584 compared to control samples of cells grown in 50% uninoculated *C. acnes* growth medium (Fig. 3A). The differential gene expression analysis provided several insights into the mechanisms that could be responsible for the reduced virulence observed in the aforementioned experiments as there were several important virulence factors encoding genes downregulated. Specifically, almost all of T3SS encoding genes were significantly downregulated, including all three exotoxins *(exoT, exoU*, and *exoY*) and the master regulator of the T3SS, *exsA* (Figs. 3A and 3B)^47^. The T3SS is a crucial virulence factor against eukaryotic cells and *P. aeruginosa* mutants lacking the T3SS displayed a significantly reduced virulence in several animal models including in the *G. mellonella* burn wound model^41,48^. Significant down-regulation of most of the genes encoding for the T3SS indicates that this effect is most likely responsible for the reduced virulence seen in the *G. mellonella* model. The evidence that the master regulator of the T3SS was also downregulated indicates that the specific mechanism of downregulation most likely lies upstream of this component of the T3SS regulatory pathway. These findings were further supported by the KEGG pathway analysis performed on the downregulated genes, which showed that bacterial secretion system encoding genes was one of the most represented gene groups (Supplementary Tables 2.1, 2.2, Supplementary Fig. 2.1, 2.2).

Certain environmental conditions are known to influence the expression of the T3SS, most notably calcium concentration, glucose availability and metabolic stress in general^49–51^. Within the RNA-Seq data set several of the downregulated genes are associated with the glucose uptake in *P. aeruginosa*. *gltF-gltG-gltK* which encodes the primary ABC transporter responsible for glucose membrane transport were significantly downregulated^52,53^. This is in agreement with previous data demonstrating the down-regulation of these uptake systems in low glucose conditions^54,55^. *gnuT* and *PA14_37250* genes involved in glucose and lactate uptake were also significantly downregulated^56^. *C. acnes* is a glucose fermenting microorganism^57,58^. This suggests that *C. acnes* could be outcompeting *P. aeruginosa* for glucose within the wound microenvironment, therefore having a negative impact on pathogenicity without impacting *P. aeruginosa* growth, as *P. aeruginosa* can readily switch between carbon sources with little effect on overall growth^59^. In addition, glucose is known to enhance *P. aeruginosa* biofilm formation suggesting a potential mechanism for the reduced biofilm phenotype^60^.

### The effects of *C. acnes* CCUG 38584 on *P. aeruginosa* PA14 are glucose availability dependent

**Fig. 4:**
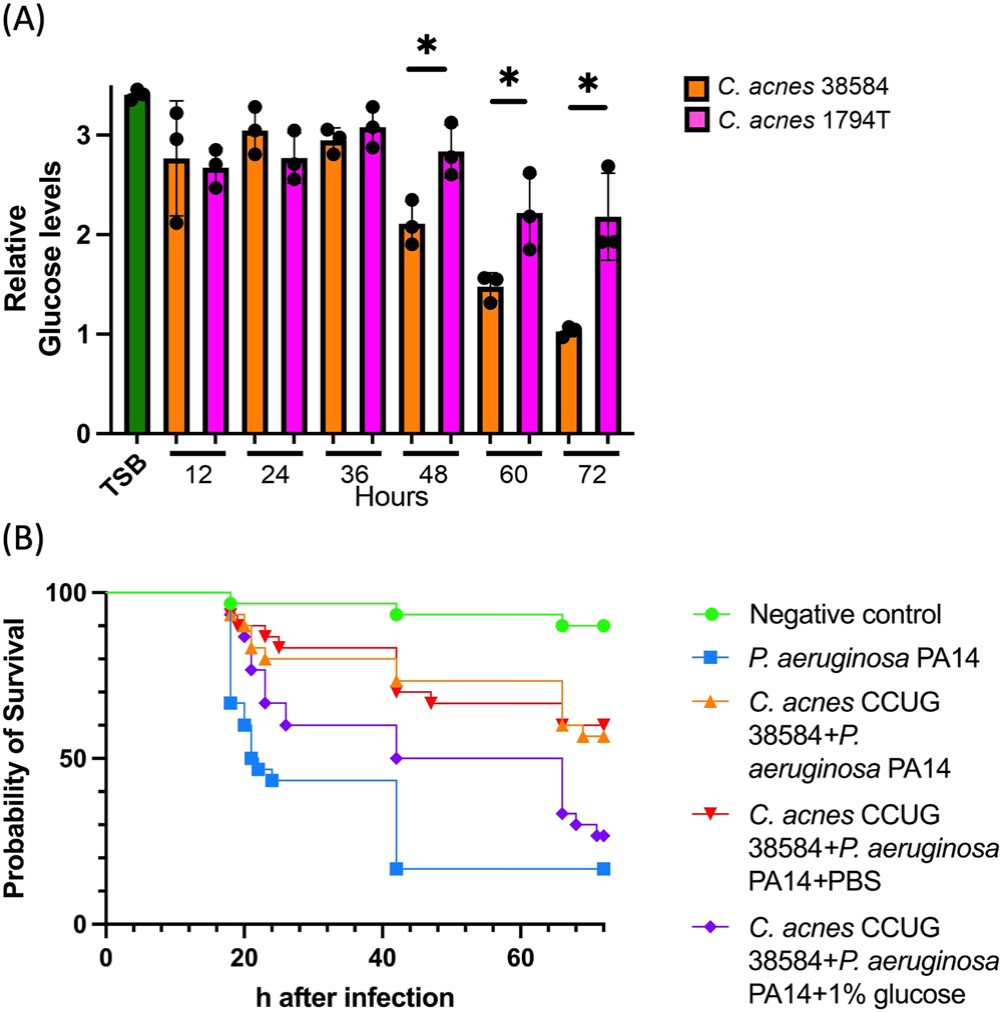
The role of glucose in the *C. acnes* antivirulence effect. **A** Glucose content quantification in the supernatants of *C. acnes* CCUG 38584 and CCUG 1794T, demonstrating a declining glucose concentration over the 72 h observation of *C. acnes* growth. There was no significant difference between the *C. acnes* strains glucose consumption observed until the 48 h mark. At 48 h, 60 h and 72 h CCUG 38584 depleted glucose in the media significantly more than *C. acnes* CCUG 1794T, unpaired Student’s *t* test *p* value < 0.05, three biological replicates with two advanced technical replicates were analysed. Error Bars are Standard Deviation. **B** Survival curve of in vivo *P. aeruginosa* PA14 burn infection with *C. acnes* CCUG 38584 treatment with topically supplemented glucose, *n* = 30, 3 biological replicates with 10 larvae per experimental group. Log-rank (Mantel-Cox) test was performed to assess the statistical significance with Bonferroni-correction. CCUG 38584 + PA14 + PBS versus CCUG 38584 + PA14 + 1% glucose, *p* value < 0.05.

The hypothesis that competition for glucose is responsible for the reduced pathogenicity of *P. aeruginosa* in a wound seeded with *C. acnes* does not explain why some strains of *C. acnes* appear to have a greater effect on pathogenicity than others, unless the rate of glucose fermentation varied significantly between *C. acnes* strains. In order to test this hypthesis, the *C. acnes* strain with the highest (*C. acnes* CCUG 38584) and the lowest antivirulence effect (*C. acnes* CCUG 1794T*)* were selected and glucose concentration was monitored over time (Fig. 4A). *C. acnes* CCUG 38584 depleted glucose from the media at a faster rate than *C. acnes* CCUG 1794T with a significant difference observed at 48 h, 60 h and 72 h. There was no significant difference observed in the growth of these two strains confirming that the observed probiotic effects were not due to a reduced growth rate between the strains (Supplementary Figure 1). This aligns with our previous findings of the antivirulence effect variability between these two strains and supports the glucose dependant therapeutic effect hypothesis.

To further challenge the implicated importance of glucose scavenging in the *C. acnes* therapeutic effect, it was hypothesised that it should be possible to mitigate the effect through the exogenous application of glucose to the co-colonised wound. The *G. mellonella* survival assay was repeated with topical supplementation with a 1% glucose solution. Seeding of the wound with *C. acnes* CCUG 38584 prior to infection with *P. aeruginosa* PA14 resulted in the anticipated increase in survival. However, groups topically supplemented with 1% glucose had an increased virulence comparable to that seen with a *P. aeruginosa* PA14 only infection, while topical supplementation with a no-glucose control solution had no negative impact on the *C. acnes* therapeutic effect (Fig. 4B). This supports the hypothesis that glucose competition is one of the primary mechanisms involved in the *P. aeruginosa* PA14 in vivo mortality reduction by *C. acnes*.

## Discussion

Wound care and management is a serious health issue worldwide, an issue that will only be exacerbated in the coming decades as a result of the growing ageing population^2,18^. One of the most commonly isolated wound pathogens is *P. aeruginosa*^61^. *P. aeruginosa* infected wounds are notoriously challenging to treat due to a wide range of virulence factors that it utilises, its metabolic flexibility and its capacity to overcome antibiotic challenge^62,63^. Therefore, there is an urgent need for alternative treatments for this pathogen.

The skin-gut microbiota axis effect on skin homoeostasis and skin wound healing has been firmly established^64,65^. Oral gut probiotic therapy has been reported to modulate immune response both locally and systemically^66–68^. In humans, probiotic supplementation of the gut has been reported to reduce inflammation, reverse liver injury and improve wound healing in chronic diabetic ulcers^69–72^. The positive effect of probiotic supplementation of the gut microbiota has also been shown to improve wound healing in rats and human intestinal myofibroblasts^73,74^. Topical applications of several commensal bacterial species have also been shown to influence wound infection and healing, such as *Lactobacillus spp* and *S. epidermidis*^42,75–78^. In the present study, we demonstrated that seeding a wound with *C. acnes* prior to the topical infection by *P. aeruginosa* PA14 significantly improves the survival of *G. mellonella* larvae (Fig. 1). One of the primary reasons for the reduction in in vivo mortality could be a reduction in the bioburden of *P. aeruginosa*. However, when tested, there was no effect of the supernatant or co-culture on the growth of *P. aeruginosa*, which implied that bacteriocins and acid production by *C. acnes* or direct contact mediated interspecies competition are unlikely to be responsible for antivirulence effect on *P. aeruginosa* in vivo. Furthermore, *C. acnes* supernatant and co-culture with *C. acnes* significantly decreases *P. aeruginosa* biofilm production (Fig. 2). Biofilm formation is one of the most important *P. aeruginosa* PA14 virulence factors, which allows it to evade the host immune system and establish persistent wound infections^79^. The reduction of biofilm formation of *P. aeruginosa* in the presence of *C. acnes* supernatant could also be a contributing factor to the reduction of in vivo mortality^80^.

In order to gain further insight into the underlying mechanism of the *C. acnes* effect on *P. aeruginosa*, differential gene expression analysis was performed on *P. aeruginosa* in the presence of *C. acnes* cell-free supernatant. Among the differentially expressed genes, the expression of genes encoding for the T3SS, the primary eukaryotic toxin delivery system of *P. aeruginosa*, was significantly downregulated. This likely explains the significant reduction in virulence as the T3SS is known to play a key role in *P. aeruginosa* pathogenicity and disrupting it has been previously shown in the model to significantly decrease pathogenicity^41,48^. Additionally, several genes associated with glucose acquisition were down-regulated including genes ecoding the high-affinity glucose transporter GltFGK and glucose uptake response regulator GltR as well as porin protein-encoding genes such as *oprB*. Several studies have demonstrated the link between glucose availability and the activity of the T3SS, with a disruption in glucose acquisition systems leading to a repression of the T3SS^49,51,54,55,81^. In addition to that, biofilm formation in *P. aeruginosa* has been reported to be glucose dependent, which would explain the reduced biofilm formation observed^60^.

Glucose is one of the preferred carbon sources for many microorganisms and as such would be a high-value commodity in the wound microenvironment^82^. *C. acnes* preferred carbon sources are glycerol and glucose which are readily available in the wound mircoenvironment^57,58,83^. This is particularly important in a burn wound setting as trauma-induced insulin resistance is known to significantly increase the levels of glucose in the wound microenvironment^84^. We demonstrate that the *C. acnes* strain CCUG 38584 which exhibited therapeutic effects against *P. aeruginosa* ferments glucose at a higher rate than the non-therapeutic CCUG 1794T, which implies that *C. acnes* CCUG 38584 is a more potent glucose scavenger in the wound (Fig. 4A). *P. aeruginosa* has a remarkable carbon source plasticity and can readily switch between carbon sources with limited effect on growth, explaining why co-culture with *C. acnes*, depleting the media of glucose, has no impact on *P. aeruginosa* growth^59^. Furthermore, the therapeutic effect of *C. acnes* CCUG 38584 was reversed upon topical supplementation of the wound with glucose (Fig. 4B). This supports the hypothesis that carbon-source competition between commensal bacteria and wound pathogens may be a potent alternative wound treatment vector as well as a prophylaxis of wound infections.

A limitation of this study is that it was performed in the *G. mellonella* burn wound model. However, this model has previously been shown to recapitulate many of the hallmarks of human burn wound trauma and is robust model for the screening and development of novel wound therapeutics^41–44^. It is also worth noting that performing the screening and characterisation described in this study in a mouse or rabbit burn wound model would be extremely difficult due to the numbers of animals required and the severity of the procedures involved. Future work will focus on further validating our findings in higher eukaryotic models such as mice and exploring the impact of *C. acnes* on other wound types such as diabetic ulcers.

In conclusion, this study has identified *C. acnes* as a potential wound probiotic, which was capable of reducing *P. aeruginosa* virulence in vivo and limiting biofilm formation. Moreover, it demonstrates a proof of principle that competitive exclusion by carbon-source competition could be an effective platform on which to develop alternative therapeutic avenues for phophylaxis and treatment of wound infections.

## Methods

### Bacterial strains growth assay

*P. aeruginosa* (PA14) and *C. acnes* (CCUG 38584, CCUG 1794T, CCUG 6369, CCUG 48370) were used. *P. aeruginosa* PA14 is a wound isolate and is a reference strain^85^. *C. acnes* CCUG 38584 belongs to phylotype I and its isolation origin is unknown^86,87^. *C. acnes* CCUG 1794T is a human facial acne isolate, phylotype IA1^57^. *C. acnes* CCUG 6369 belongs to phylotype II and was isolated from a human subcutaneous abscess^88^. *C. acnes* CCUG 48370 is a vaginal discharge isolate and its phylotype is unknown^45^. Strain information is also available in Supplementary Table 1. *P. aeruginosa* was grown in LB Broth (Miller, Fisher BioReagents BP1426-2) overnight at 37 °C at 180 rpm and on LB Agar (Miller, Fisher BioReagents Microbiology Media) plates overnight at 37 °C. *C. acnes* was grown in tryptic soy broth (TSB, NutriSelect Plus) and on blood agar plates for 48–72 h at 37 °C. Blood agar plates were prepared by supplementing tryptic soy agar (NutriSelect Plus) with defibrinated horse blood (Thermo Scientific Oxoid, SR0050C) in a ratio of 19:1. *C. acnes* was grown anaerobically in a hermetic chamber with an AnaeroGen 2.5 L Sachet (Thermo Scientific Oxoid).

### Growth and biofilm assay

Overnight cultures of *P. aeruginosa* PA14 were adjusted to OD = 0.1 at 600 nm. OD-adjusted bacteria were then washed by centrifugation at 8000 rpm for 3 mins, disposing of the supernatant and resuspending the bacterial pellet in fresh media 3 times. Washed culture was pipetted into a 96-well plate with 200 µl for positive control and 100 µl for the experimental conditions. PA14 was tested against 100 µl TSB broth and 100 µl of *C. acnes* strains cell-free supernatant. *C. acnes* strains supernatant was obtained by centrifuging the 48–72 h cultures for 10 mins at 5000 rpm and filter sterilising the supernatant with 0.2 nm filter. The set-up 96-well plate was incubated overnight in the Clariostar plate reader at 37 °C at 200 rpm to obtain a growth measurement. Upon the completion of the growth curve, the 96-well plate was taken out of the plate reader and the contents of each well were pipetted out. Wells were washed with 250 µl of distilled water 3 times. After washing, wells were stained with 220 µl of 1% crystal violet solution for 10 mins. Wells were then washed with 250 µl of distilled water to get rid of excess stain 5 times. The 96-well plate was then left to dry for 2 h. After the plate dried, 200 µl of 99% ethanol was added to each well and left to de-stain for 3 h. The de-stained well absorbance was read at 600 nm in the Clariostar plate reader.

### *P. aeruginosa* cellular co-culture with *C. acnes*

*P. aeruginosa* strains PA14, and *C. acnes* strains CCUG 1794 and *C. acnes* CCUG 38584 were grown (either alone or in combination) in an artifical sebum model which was prepared and inoculated as previously described^89^. Following incubation at 37 °C under aerobic conditions (for 24 h and 48 h), the number of CFU was quantified by plating on Difco Pseudomonas Isolation Agar (PIA; BD Diagnostics) and Reinforced Clostridual Medium (RCM; Oxoid). PIA plates were incubated aerobically and RCM plates anaerobically (both at 37 °C). Biofilm quantification by plating was performed as previously described, artifical sebum was placed into tubes with 10 mL PS, the sessile cells were removed by three cycles of vortexing and sonication (Branson 3510; Branson Ultrasonics Corp., Danbury, CT) and the number of CFU/biofilm was determined by plating the resulting suspensions of Difco Pseudomonas Isolation Agar (PIA; BD Diagnostics).

### Animal acquisition and preparation

*Galleria mellonella* were obtained from LiveFood UK Ltd. (Somerset, United Kingdom). Only 6th instar larvae were used for experiments, which is the life stage at which they do not require feeding and weigh approximately 200 mg. Prior to use, larvae were stored at 4 °C. Before the experiment, larvae were sorted into Petri dishes lined with filter paper (Whatman, Fisher, United Kingdom). 10 caterpillars per dish were allowed and then stored at 4 °C until use.

### In vivo burn infection and treatment assay

70% ethanol was used to sterilise the larval body surface. Petri dishes were left open in a sterile environment to allow for the ethanol to evaporate after sterilisation. The burn was induced by using a heated steel element embedded in insulating material to consistently achieve a burn area of approximately 2 mm^2^. The burn instrument was heated in the middle flame of the Bunsen burner until it was red/white-hot and applied to the middle segment of *G. mellonella* back for 4 s. The burn wound was colonised with *C. acnes*, by picking up a few colonies from the blood agar plate with a pipette tip and gently brushing it against the burn wound surface. Immediately afterwards, 10 µl of overnight culture of PA14 grown to ~OD_600_ = 3 was applied topically to the wound. Any larva that showed a major haemolymph loss or protruding insides after the procedure was immediately euthanized by placing it at −20 °C for at least 20 mins to minimise suffering. Post-procedure the invertebrates were incubated at 37 °C for 72 h to monitor. Mortality was recorded upon the complete loss of motility and melanisation of the larval body^41^.

For glucose treatment experiments, 1 h post *P. aeruginosa* PA14 inoculation into the wound, wounds were treated with 10 µl of either 1% glucose solution or PBS. Any larva that showed a major haemolymph loss or protruding insides after the procedure was immediately euthanized by placing it at −20 °C for at least 20 mins to minimise suffering. Post-procedure the invertebrates were incubated at 37 °C for 72 h to monitor. Mortality was recorded upon the complete loss of motility and melanisation of the larval body^41^.

### RNA extraction and sequencing

*P. aeruginosa* PA14 was grown in the overnight tube at 37 °C at 180 rpm. Cultures were then adjusted to OD = 0.1 with LB and added in proportion 1:1 to TSB or the supernatant of *C. acnes* CCUG 38584 to be grown to OD_600_ = 0.6–0.7 at 37 °C at 180 rpm. Upon reaching the desired optical density, 1 ml of the cultures was aliquoted and spun down at 5000 rpm for 10 mins, the supernatant was discarded, and the pellets were resuspended in the RNAlater buffer (ThermoFischer). The resuspended cells were stored in the buffer at 4 °C overnight. RNA extraction procedure was performed with RNeasy Mini Kit (Qiagen). Extracted RNA was quantified using Nanodrop. The quality of the extraction was assessed with Bioanalyser. Extracted RNA samples were stored at −20 °C until shipment to the sequencing facility. The cDNA synthesis and Illumina sequencing were performed by Microbial Genome Sequencing Centre (MiGS). Samples were DNase treated with Invitrogen DNase (RNase free). Library preparation was performed using Illumina’s Stranded Total RNA Prep Ligation with Ribo-Zero Plus kit and IDT for Illumina indices. Sequencing was performed on a NextSeq2000 giving 2x50bp reads.

Raw fastq files were obtained from the sequencing facility. Adapter and quality trimming were performed by MiGS using bcl2fastq. Reads and their quality was assessed with *fastqc* and visualised through *multiqc*^90,91^. Read mapping was performed with *hisat2* with ‘--very-sensitive’ parameter^92^. Read quantification was performed with *featureCounts* available within the *Subread* package^93^. Read normalisation was performed using the *edgeR* package in R with the Trimmed Mean of M values algorithm^94^. Differential expression analysis was performed using *edgeR*’s Quasi-Linear F-Test (qlfTest) functionality against treatment groups. All quantified genes were subset by log-fold change (logFC) > |1| and *p*-value < 0.05 to create a list of differentially expressed genes. All quantified genes were visualised in a volcano plot in *ggplot2 R* package. An additional table demonstrating the subset of genes with (logFC) > |2| and p-value < 0.05 can be found in Supplementary Table 3. The differentially expressed genes were additionally analysed with KEGG pathway analysis (FUNAGE-Pro) and the results were made available in the supplementary materials (Supplementary Tables 2.1, 2.2, Supplementary Figs. 2.1, 2.2).

### Glucose content quantification of *C. acnes* supernatants

*C. acnes* strains were grown in 15 ml of tryptic soy broth (TSB, NutriSelect Plus) in a 50 ml Falcon tube for 72 h at 37 °C. *C. acnes* were grown anaerobically in a hermetic chamber with an AnaeroGen™ 2.5 L Sachet (Thermo Scientific™ Oxoid™). Every 12 h 1 ml of the culture was pipetted out into an Eppendorf tube and frozen at −20 °C. At 0 h 1 ml of TSB was aliquoted and frozen as well. Once the 72 h sample was collected, the glucose content was measured with Glucose Colorimetric kit (Thermo-Fischer Scientific) in accordance with the protocol provided in the kit. Samples with the highest OD at 24 h were removed from the pool.

### Statistical analysis

For survival curves, the Log Rank statistical test was used to determine the significance of the findings and the values were corrected for multiple comparisons using the Bonferroni correction. Unpaired Student’s *t* test was performed on glucose content and biofilm formation data. Two-way ANOVA was performed for the growth curve statistical significance assessment. For the gene differential expression p values were generated by using the Quasi-linear F-test.

### Supplementary information

Supplementary Material

## Data Availability

The RNA-seq datasets produced in this study are available at the National Center for Biotechnology Information Gene Expression Omnibus public database under accession number GSE236405.
