# Supplementary material for: Carbon source competition within the wound microenvironment can significantly influence infection progression
Source: NPJ Biofilms Microbiomes. 2024 Jun 25;10:52. doi: 10.1038/s41522-024-00518-4 (PMC11199515; doi:10.1038/s41522-024-00518-4)

Supplementary materials.

Supplementary table 1. The table of *C. acnes* strains used in this study and their origins and phylotypes**.**

	**Origin**	**Phylotype**
***C. acnes* CCUG 38584**	Unknown	I
***C. acnes* CCUG 1794T**	Human facial acne isolate	IA1
***C. acnes* CCUG 6369**	Human subcutaneous abscess	II
***C. acnes* CCUG 48370**	Vaginal discharge isolate	Unknown

Supplementary Table 2.1. The gene set enrichment analysis of up-regulated differentially expressed genes of P. aeruginosa PA14 (annotated with Lee et al., 2006 genome) in the presence of cell-free supernatant of C. acnes

ClassID	Class	Description	score; hits/class size; p-value; gene list
00190 Oxidative phosphorylation [PATH:ko00190]	KEGG	ko00190	0;6/ 44;1.4e-04;PA14_13040,PA14_34320,PA14_47160,PA14_47180,PA14_47190,PA14_47210
00640 Propanoate metabolism [PATH:ko00640]	KEGG	ko00640	0;3/ 29;9.6e-03;PA14_53940,PA14_53950,PA14_53980
02010 ABC transporters [PATH:ko02010]	KEGG	ko02010	0;6/147;4.8e-02;PA14_09300,PA14_09320,PA14_20170,PA14_34260,PA14_36220,PA14_45370
01053 Biosynthesis of siderophore group nonribosomal peptides [PATH:ko01053]	KEGG	ko01053	2;3/ 6;2.6e-05;PA14_09220,PA14_09270,PA14_09280
Q	COG	METABOLISM; Secondary metabolites biosynthesis, transport, and catabolism	0; 4/ 58;0.040;PA14_09220,PA14_09270,PA14_09280,PA14_48060
C	COG	METABOLISM; Energy production and conversion	0;11/227;0.027;PA14_13030,PA14_13040,PA14_33860,PA14_39540,PA14_40510,PA14_47160,PA14_47180,PA14_47190,PA14_47210,PA14_53950,PA14_53970
COG2226	eggNOG_COG	methyltransferase	0;2/9;5.5e-04;PA14_09270,PA14_09280
COG3433	eggNOG_COG	isochorismatase activity	3;2/3;2.1e-05;PA14_09270,PA14_09280
COG1020	eggNOG_COG	D-alanine [D-alanyl carrier protein] ligase activity	1;2/5;1.0e-04;PA14_09270,PA14_09280
GO:0005887	GO	integral component of plasma membrane	0; 7/145;2.4e-02;PA14_09680,PA14_35620,PA14_40510,PA14_43490,PA14_47160,PA14_47180,PA14_47190
GO:0009697	GO	salicylic acid biosynthetic process	2; 2/ 3;4.1e-05;PA14_09210,PA14_09220
GO:0006865	GO	amino acid transport	0; 3/ 53;4.4e-02;PA14_17740,PA14_34260,PA14_36220
GO:0030170	GO	pyridoxal phosphate binding	0; 4/ 67;2.5e-02;PA14_03450,PA14_06890,PA14_06920,PA14_70160
GO:0015990	GO	electron transport coupled proton transport	2; 4/ 10;5.6e-06;PA14_40510,PA14_47160,PA14_47180,PA14_47190
GO:0042626	GO	ATPase-coupled transmembrane transporter activity	0; 4/ 23;3.2e-04;PA14_09300,PA14_09320,PA14_30550,PA14_34260
GO:0009319	GO	cytochrome o ubiquinol oxidase complex	9; 2/ 2;0.0e+00;PA14_47160,PA14_47180
GO:0008152	GO	metabolic process	0; 2/ 7;8.5e-04;PA14_09270,PA14_09280
GO:0022857	GO	transmembrane transporter activity	0; 7/140;2.0e-02;PA14_13530,PA14_13560,PA14_30550,PA14_36220,PA14_43490,PA14_52940,PA14_66510
GO:0016682	GO	oxidoreductase activity, acting on diphenols and related substances as donors, oxygen as acceptor	9; 2/ 2;0.0e+00;PA14_13030,PA14_47190
GO:0004457	GO	lactate dehydrogenase activity	9; 2/ 2;0.0e+00;PA14_33860,PA14_63090
GO:0042864	GO	pyochelin biosynthetic process	9; 2/ 2;0.0e+00;PA14_09210,PA14_09220
GO:0009055	GO	electron transfer activity	0; 4/ 78;4.4e-02;PA14_13030,PA14_39540,PA14_47160,PA14_47180
GO:0019629	GO	propionate catabolic process, 2-methylcitrate cycle	1; 2/ 5;3.1e-04;PA14_53940,PA14_53980
GO:0009060	GO	aerobic respiration	1; 3/ 10;1.4e-04;PA14_47160,PA14_47180,PA14_47190
GO:0032259	GO	methylation	0; 3/ 36;1.5e-02;PA14_06660,PA14_53910,PA14_73140
GO:0004129	GO	cytochrome-c oxidase activity	1; 4/ 13;2.5e-05;PA14_40510,PA14_47180,PA14_47190,PA14_47210
GO:0008827	GO	cytochrome o ubiquinol oxidase activity	9; 3/ 3;0.0e+00;PA14_47160,PA14_47180,PA14_47210
GO:0006807	GO	nitrogen compound metabolic process	0; 3/ 14;5.0e-04;PA14_17730,PA14_64660,PA14_64670
GO:0019516	GO	lactate oxidation	9; 2/ 2;0.0e+00;PA14_33860,PA14_63090
GO:0015453	GO	oxidoreduction-driven active transmembrane transporter activity	9; 2/ 2;0.0e+00;PA14_47160,PA14_47180
GO:0006119	GO	oxidative phosphorylation	1; 3/ 9;9.1e-05;PA14_13030,PA14_13040,PA14_40510
GO:0006099	GO	tricarboxylic acid cycle	0; 3/ 28;6.5e-03;PA14_53950,PA14_53970,PA14_54000
GO:0005507	GO	copper ion binding	0; 3/ 17;9.9e-04;PA14_37810,PA14_40510,PA14_47210
GO:0016021	GO	integral component of membrane	0;22/720;4.4e-02;PA14_09300,PA14_09320,PA14_09370,PA14_13030,PA14_13040,PA14_13520,PA14_13530,PA14_13560,PA14_17740,PA14_20040,PA14_20170,PA14_28050,PA14_34260,PA14_40540,PA14_44520,PA14_47150,PA14_47210,PA14_49130,PA14_52940,PA14_64680,PA14_66510,PA14_72960
GO:0005886	GO	plasma membrane	0;28/845;9.1e-03;PA14_09300,PA14_09320,PA14_09370,PA14_10270,PA14_13030,PA14_13040,PA14_13520,PA14_13530,PA14_17740,PA14_20040,PA14_20150,PA14_20170,PA14_28050,PA14_33860,PA14_34260,PA14_36220,PA14_40510,PA14_40540,PA14_43490,PA14_44520,PA14_47150,PA14_47210,PA14_49130,PA14_52940,PA14_63090,PA14_64680,PA14_66510,PA14_72960
GO:0070469	GO	respirasome	0; 2/ 10;2.6e-03;PA14_47190,PA14_47210
GO:0005615	GO	extracellular space	0; 2/ 7;8.5e-04;PA14_30570,PA14_48060
GO:0019679	GO	propionate metabolic process, methylcitrate cycle	1; 3/ 7;3.0e-05;PA14_53950,PA14_53970,PA14_54000
GO:0019646	GO	aerobic electron transport chain	2; 4/ 10;5.6e-06;PA14_13030,PA14_40510,PA14_47160,PA14_47180
GO:0070069	GO	cytochrome complex	1; 2/ 5;3.1e-04;PA14_13030,PA14_40510
GO:0016151	GO	nickel cation binding	1; 3/ 7;3.0e-05;PA14_64650,PA14_64660,PA14_64670
GO:0009486	GO	cytochrome bo3 ubiquinol oxidase activity	9; 3/ 3;0.0e+00;PA14_47160,PA14_47180,PA14_47190
GO:0042597	GO	periplasmic space	0; 5/109;4.6e-02;PA14_30550,PA14_30570,PA14_39540,PA14_64690,PA14_72360
IPR023615	IPR	Cytochrome c oxidase, subunit I, copper-binding site	0;2/ 7;1.1e-03;PA14_40510,PA14_47190
IPR015421	IPR	Pyridoxal phosphate-dependent transferase, major domain	0;3/57;4.5e-02;PA14_03450,PA14_06920,PA14_70160
IPR012133	IPR	Alpha-hydroxy acid dehydrogenase, FMN-dependent	9;2/ 2;0.0e+00;PA14_33860,PA14_63090
IPR029063	IPR	S-adenosyl-L-methionine-dependent methyltransferase	0;5/68;7.1e-03;PA14_09270,PA14_09280,PA14_19400,PA14_53910,PA14_73140
IPR015424	IPR	Pyridoxal phosphate-dependent transferase	0;3/57;4.5e-02;PA14_03450,PA14_06920,PA14_70160
IPR029058	IPR	Alpha/Beta hydrolase fold	0;3/52;4.0e-02;PA14_09230,PA14_19100,PA14_29340
IPR037396	IPR	FMN hydroxy acid dehydrogenase domain	9;2/ 2;0.0e+00;PA14_33860,PA14_63090
IPR008259	IPR	FMN-dependent alpha-hydroxy acid dehydrogenase, active site	9;2/ 2;0.0e+00;PA14_33860,PA14_63090
IPR020920	IPR	L-lactate dehydrogenase, bacterial	9;2/ 2;0.0e+00;PA14_33860,PA14_63090
IPR006726	IPR	Para-hydroxybenzoic acid efflux pump subunit AaeB/ fusaric acid resistance protein	1;2/ 4;2.2e-04;PA14_13530,PA14_52940
IPR000262	IPR	FMN-dependent dehydrogenase	9;2/ 2;0.0e+00;PA14_33860,PA14_63090
IPR036927	IPR	Cytochrome c oxidase-like, subunit I superfamily	0;2/ 7;1.1e-03;PA14_40510,PA14_47190
IPR013217	IPR	Methyltransferase type 12	2;2/ 3;6.5e-05;PA14_09270,PA14_09280
IPR005814	IPR	Aminotransferase class-III	0;3/14;8.4e-04;PA14_03450,PA14_06920,PA14_70160
IPR000883	IPR	Cytochrome c oxidase subunit I	0;2/ 7;1.1e-03;PA14_40510,PA14_47190
IPR015422	IPR	Pyridoxal phosphate-dependent transferase domain 1	0;3/53;4.1e-02;PA14_03450,PA14_06920,PA14_70160
IPR023616	IPR	Cytochrome c oxidase-like, subunit I domain	0;2/ 7;1.1e-03;PA14_40510,PA14_47190
Chaperone	KEYWORDS	keyword	0; 3/ 26;0.00651;PA14_64650,PA14_64660,PA14_64670
Ligase	KEYWORDS	keyword	1; 2/ 6;0.00077;PA14_09270,PA14_09280
Cell inner membrane	KEYWORDS	keyword	0;18/366;0.00077;PA14_13030,PA14_13040,PA14_20040,PA14_20170,PA14_33860,PA14_35620,PA14_44520,PA14_45370,PA14_47150,PA14_47160,PA14_47180,PA14_47190,PA14_47210,PA14_49130,PA14_63090,PA14_64680,PA14_66510,PA14_72960
Lipid biosynthesis	KEYWORDS	keyword	2; 2/ 3;0.00012;PA14_53910,PA14_73140
operon_0673	operons	CP000438.1|gene1430|PA14_17740|ycjJ	9;2/ 2;0.0e+00;PA14_17740,PA14_17760
operon_0818	operons	CP000438.1|gene1734|PA14_21570	9;4/ 4;0.0e+00;PA14_21570,PA14_21580,PA14_21590,PA14_21600
operon_1305	operons	CP000438.1|gene2777|PA14_34260|metI-1	1;2/ 6;1.9e-04;PA14_34260,PA14_34320
operon_1779	operons	CP000438.1|gene3813|PA14_47150|cyoE	9;5/ 5;0.0e+00;PA14_47150,PA14_47160,PA14_47180,PA14_47190,PA14_47210
operon_2060	operons	CP000438.1|gene4380|PA14_53920	9;4/ 4;0.0e+00;PA14_53920,PA14_53940,PA14_53950,PA14_53970
operon_1701	operons	CP000438.1|gene3659|PA14_45260	0;2/10;1.0e-03;PA14_45260,PA14_45370
operon_0788	operons	CP000438.1|gene1681|PA14_20920	1;3/ 9;2.7e-05;PA14_20920,PA14_20940,PA14_20950
operon_0269	operons	CP000438.1|gene545|PA14_06890	9;4/ 4;0.0e+00;PA14_06890,PA14_06900,PA14_06920,PA14_06930
operon_2452	operons	CP000438.1|gene5277|PA14_64650|ureE	9;4/ 4;0.0e+00;PA14_64650,PA14_64660,PA14_64670,PA14_64680
operon_0520	operons	CP000438.1|gene1084|PA14_13520	9;2/ 2;0.0e+00;PA14_13520,PA14_13530
operon_1050	operons	CP000438.1|gene2228|PA14_27600	9;3/ 3;0.0e+00;PA14_27600,PA14_27610,PA14_27620
operon_0344	operons	CP000438.1|gene744|PA14_09210|pchA	4;3/ 4;2.5e-07;PA14_09210,PA14_09220,PA14_09230
operon_2389	operons	CP000438.1|gene5152|PA14_63090|lldD	9;2/ 2;0.0e+00;PA14_63090,PA14_63100
operon_0347	operons	CP000438.1|gene756|PA14_09370	9;2/ 2;0.0e+00;PA14_09370,PA14_09380
operon_0500	operons	CP000438.1|gene1045|PA14_13030|cioA	9;3/ 3;0.0e+00;PA14_13030,PA14_13040,PA14_13050
operon_0762	operons	CP000438.1|gene1616|PA14_20150|nosL	1;2/ 6;1.9e-04;PA14_20150,PA14_20170
operon_0346	operons	CP000438.1|gene749|PA14_09270|pchE	6;5/ 7;8.0e-10;PA14_09270,PA14_09280,PA14_09290,PA14_09300,PA14_09320
PF00115	Pfam	Cytochrome C and Quinol oxidase polypeptide I	0;2/ 7;7.1e-04;PA14_40510,PA14_47190
PF01070	Pfam	FMN-dependent dehydrogenase	9;2/ 2;0.0e+00;PA14_33860,PA14_63090
PF04632	Pfam	Fusaric acid resistance protein family	1;2/ 4;1.4e-04;PA14_13530,PA14_52940
PF00202	Pfam	Aminotransferase class-III	0;3/14;4.6e-04;PA14_03450,PA14_06920,PA14_70160
PF08242	Pfam	Methyltransferase domain	2;2/ 3;5.4e-05;PA14_09270,PA14_09280

Supplementary Table 3. Differentially expressed genes of P. aeruginosa PA14 in the presence of the C. acnes CCUG 38584, p value < 0.05, Log-fold change > |2|.

Differentially_Expressed_Genes																				
**table.rn**	table.Chr	table.Start	table.End	table.Strand	table.Length	table.logFC	table.logCPM	table.F	table.PValue	table.FDR	adjust.method	comparison	test	attributes	attributes.ID	attributes.Name	attributes.Note	attributes.gbkey	attributes.gene_biotype	attributes.locus_tag
**gene-PA14_52960**	CP000438.1	4695869	4696135	+	267	5.15939561374669	6.18381877822789	96.2249029026831	4.11708469553171E-06	0.000659534095531427	BH	group2	glm	ID=gene-PA14_52960;Name=PA14_52960;Note=corresponds to Pseudomonas aeruginosa strain PAO1 locus PA0874;gbkey=Gene;gene_biotype=protein_coding;locus_tag=PA14_52960	gene-PA14_52960	PA14_52960	corresponds to Pseudomonas aeruginosa strain PAO1 locus PA0874	Gene	protein_coding	PA14_52960
**gene-PA14_01990**	CP000438.1	182682	182867	+	186	4.34273679031422	5.34833109181432	127.556655085521	1.25956239551406E-06	0.000363194816746479	BH	group2	glm	ID=gene-PA14_01990;Name=PA14_01990;Note=corresponds to Pseudomonas aeruginosa strain PAO1 locus PA0160;gbkey=Gene;gene_biotype=protein_coding;locus_tag=PA14_01990	gene-PA14_01990	PA14_01990	corresponds to Pseudomonas aeruginosa strain PAO1 locus PA0160	Gene	protein_coding	PA14_01990
**gene-PA14_53940**	CP000438.1	4782285	4783181	+	897	3.5616561778769	7.63838910579401	229.221629334703	1.01091650209627E-07	0.000130422998554971	BH	group2	glm	ID=gene-PA14_53940;Name=prpB;Note=corresponds to Pseudomonas aeruginosa strain PAO1 locus PA0796;gbkey=Gene;gene=prpB;gene_biotype=protein_coding;gene_synonym=bcpA;locus_tag=PA14_53940	gene-PA14_53940	prpB	corresponds to Pseudomonas aeruginosa strain PAO1 locus PA0796	Gene	protein_coding	PA14_53940
**gene-PA14_53980**	CP000438.1	4787257	4788444	+	1188	3.18386150956335	6.36988823973696	243.897783135127	7.71213801589264E-08	0.000130422998554971	BH	group2	glm	ID=gene-PA14_53980;Name=PA14_53980;Note=corresponds to Pseudomonas aeruginosa strain PAO1 locus PA0793;gbkey=Gene;gene_biotype=protein_coding;locus_tag=PA14_53980	gene-PA14_53980	PA14_53980	corresponds to Pseudomonas aeruginosa strain PAO1 locus PA0793	Gene	protein_coding	PA14_53980
**gene-PA14_13050**	CP000438.1	1120390	1120560	+	171	3.07710882672473	2.13361601281536	93.6179449585674	4.6148328938693E-06	0.000719290305376872	BH	group2	glm	ID=gene-PA14_13050;Name=PA14_13050;gbkey=Gene;gene_biotype=protein_coding;locus_tag=PA14_13050	gene-PA14_13050	PA14_13050		Gene	protein_coding	PA14_13050
**gene-PA14_33860**	CP000438.1	3008839	3009990	+	1152	3.01229300585034	4.9387608331805	105.321000184261	2.82393687013371E-06	0.000525343352582617	BH	group2	glm	ID=gene-PA14_33860;Name=lldA;gbkey=Gene;gene=lldA;gene_biotype=protein_coding;locus_tag=PA14_33860	gene-PA14_33860	lldA		Gene	protein_coding	PA14_33860
**gene-PA14_53970**	CP000438.1	4784532	4787138	+	2607	3.00950321111788	8.25765705450595	205.86610326854	1.61305300399104E-07	0.000130422998554971	BH	group2	glm	ID=gene-PA14_53970;Name=PA14_53970;Note=corresponds to Pseudomonas aeruginosa strain PAO1 locus PA0794;gbkey=Gene;gene_biotype=protein_coding;locus_tag=PA14_53970	gene-PA14_53970	PA14_53970	corresponds to Pseudomonas aeruginosa strain PAO1 locus PA0794	Gene	protein_coding	PA14_53970
**gene-PA14_54000**	CP000438.1	4788579	4790063	+	1485	3.00335696448511	6.1611482571401	98.5976493998155	3.71999119232603E-06	0.000637484100716755	BH	group2	glm	ID=gene-PA14_54000;Name=prpD;Note=corresponds to Pseudomonas aeruginosa strain PAO1 locus PA0792;gbkey=Gene;gene=prpD;gene_biotype=protein_coding;locus_tag=PA14_54000	gene-PA14_54000	prpD	corresponds to Pseudomonas aeruginosa strain PAO1 locus PA0792	Gene	protein_coding	PA14_54000
**gene-PA14_53920**	CP000438.1	4781552	4782274	+	723	2.99561403588112	4.8881068924049	199.49315703053	1.84874920132567E-07	0.000130422998554971	BH	group2	glm	ID=gene-PA14_53920;Name=PA14_53920;Note=corresponds to Pseudomonas aeruginosa strain PAO1 locus PA0797;gbkey=Gene;gene_biotype=protein_coding;locus_tag=PA14_53920	gene-PA14_53920	PA14_53920	corresponds to Pseudomonas aeruginosa strain PAO1 locus PA0797	Gene	protein_coding	PA14_53920
**gene-PA14_72960**	CP000438.1	6496412	6497719	+	1308	2.88726548755851	4.12969764832593	85.3195978977162	6.77397604435932E-06	0.000849250431474353	BH	group2	glm	ID=gene-PA14_72960;Name=kgtP;gbkey=Gene;gene=kgtP;gene_biotype=protein_coding;locus_tag=PA14_72960	gene-PA14_72960	kgtP		Gene	protein_coding	PA14_72960
**gene-PA14_53950**	CP000438.1	4783274	4784401	+	1128	2.88373615608622	8.22535936384101	170.042649036663	3.68604813929283E-07	0.000155545646861789	BH	group2	glm	ID=gene-PA14_53950;Name=prpC;Note=corresponds to Pseudomonas aeruginosa strain PAO1 locus PA0795;gbkey=Gene;gene=prpC;gene_biotype=protein_coding;locus_tag=PA14_53950	gene-PA14_53950	prpC	corresponds to Pseudomonas aeruginosa strain PAO1 locus PA0795	Gene	protein_coding	PA14_53950
**gene-PA14_47150**	CP000438.1	4201706	4202596	-	891	2.756244121604	3.20559103247893	118.871411596601	1.6975134771836E-06	0.000407898342621575	BH	group2	glm	ID=gene-PA14_47150;Name=cyoE;gbkey=Gene;gene=cyoE;gene_biotype=protein_coding;locus_tag=PA14_47150	gene-PA14_47150	cyoE		Gene	protein_coding	PA14_47150
**gene-PA14_17730**	CP000438.1	1523344	1524159	+	816	2.55008843137113	3.80177682563169	205.27172656714	1.63341902097609E-07	0.000130422998554971	BH	group2	glm	ID=gene-PA14_17730;Name=PA14_17730;Note=corresponds to Pseudomonas aeruginosa strain PAO1 locus PA3598;gbkey=Gene;gene_biotype=protein_coding;locus_tag=PA14_17730	gene-PA14_17730	PA14_17730	corresponds to Pseudomonas aeruginosa strain PAO1 locus PA3598	Gene	protein_coding	PA14_17730
**gene-PA14_47180**	CP000438.1	4202942	4203571	-	630	2.44486446032351	3.95862037600671	123.908907748035	1.42432052652965E-06	0.000365993306685291	BH	group2	glm	ID=gene-PA14_47180;Name=cyoC;gbkey=Gene;gene=cyoC;gene_biotype=protein_coding;locus_tag=PA14_47180	gene-PA14_47180	cyoC		Gene	protein_coding	PA14_47180
**gene-PA14_36200**	CP000438.1	3225300	3226106	+	807	2.29459188086627	3.95651964194259	22.4669266401465	0.00105130529207479	0.0285984793367703	BH	group2	glm	ID=gene-PA14_36200;Name=PA14_36200;Note=corresponds to Pseudomonas aeruginosa strain PAO1 locus PA2204;gbkey=Gene;gene_biotype=protein_coding;locus_tag=PA14_36200	gene-PA14_36200	PA14_36200	corresponds to Pseudomonas aeruginosa strain PAO1 locus PA2204	Gene	protein_coding	PA14_36200
**gene-PA14_13030**	CP000438.1	1117899	1119365	+	1467	2.2781508572895	5.24744176070194	111.634266883977	2.21186960901736E-06	0.000455566144114397	BH	group2	glm	ID=gene-PA14_13030;Name=cioA;gbkey=Gene;gene=cioA;gene_biotype=protein_coding;locus_tag=PA14_13030	gene-PA14_13030	cioA		Gene	protein_coding	PA14_13030
**gene-PA14_02010**	CP000438.1	183147	183299	+	153	2.22755004436716	1.98866209508447	34.3015987767986	0.000239234848120273	0.00958102339659453	BH	group2	glm	ID=gene-PA14_02010;Name=PA14_02010;Note=corresponds to Pseudomonas aeruginosa strain PAO1 locus PA0161;gbkey=Gene;gene_biotype=protein_coding;locus_tag=PA14_02010	gene-PA14_02010	PA14_02010	corresponds to Pseudomonas aeruginosa strain PAO1 locus PA0161	Gene	protein_coding	PA14_02010
**gene-PA14_73140**	CP000438.1	6512693	6513877	-	1185	2.19312763956219	7.84045851683644	9.67129968350889	0.0124817360919478	0.143105709825572	BH	group2	glm	ID=gene-PA14_73140;Name=PA14_73140;Note=corresponds to Pseudomonas aeruginosa strain PAO1 locus PA5546;gbkey=Gene;gene_biotype=protein_coding;locus_tag=PA14_73140	gene-PA14_73140	PA14_73140	corresponds to Pseudomonas aeruginosa strain PAO1 locus PA5546	Gene	protein_coding	PA14_73140
**gene-PA14_47160**	CP000438.1	4202607	4202942	-	336	2.11279956460008	2.04053002364782	84.0394796131536	7.20890022399725E-06	0.000884547395570046	BH	group2	glm	ID=gene-PA14_47160;Name=cyoD;gbkey=Gene;gene=cyoD;gene_biotype=protein_coding;locus_tag=PA14_47160	gene-PA14_47160	cyoD		Gene	protein_coding	PA14_47160
**gene-PA14_47190**	CP000438.1	4203575	4205551	-	1977	2.08389284479379	5.65852146311966	100.827009677546	3.38874994688494E-06	0.000610716279490171	BH	group2	glm	ID=gene-PA14_47190;Name=cyoB;gbkey=Gene;gene=cyoB;gene_biotype=protein_coding;locus_tag=PA14_47190	gene-PA14_47190	cyoB		Gene	protein_coding	PA14_47190
**gene-PA14_21570**	CP000438.1	1870142	1870456	+	315	2.05543257111845	2.92935724782374	76.409281353383	1.06459921378086E-05	0.00120383209134788	BH	group2	glm	ID=gene-PA14_21570;Name=PA14_21570;Note=corresponds to Pseudomonas aeruginosa strain PAO1 locus PA3284;gbkey=Gene;gene_biotype=protein_coding;locus_tag=PA14_21570	gene-PA14_21570	PA14_21570	corresponds to Pseudomonas aeruginosa strain PAO1 locus PA3284	Gene	protein_coding	PA14_21570
**gene-PA14_42550**	CP000438.1	3786710	3787576	-	867	-2.00993009522234	1.95568004584087	37.0102719202214	0.000180864282908659	0.00817072960553785	BH	group2	glm	ID=gene-PA14_42550;Name=popN;gbkey=Gene;gene=popN;gene_biotype=protein_coding;gene_synonym=yopN;locus_tag=PA14_42550	gene-PA14_42550	popN		Gene	protein_coding	PA14_42550
**gene-PA14_42290**	CP000438.1	3771000	3771431	-	432	-2.03804725222504	2.44441498581296	50.7132131307269	5.46201855547245E-05	0.00366272802434995	BH	group2	glm	ID=gene-PA14_42290;Name=pscH;gbkey=Gene;gene=pscH;gene_biotype=protein_coding;gene_synonym=yscH;locus_tag=PA14_42290	gene-PA14_42290	pscH		Gene	protein_coding	PA14_42290
**gene-PA14_64950**	CP000438.1	5789189	5789848	+	660	-2.04225821193854	4.68488673195128	64.8170121697005	2.07108041780842E-05	0.00189586043960336	BH	group2	glm	ID=gene-PA14_64950;Name=pncA;gbkey=Gene;gene=pncA;gene_biotype=protein_coding;locus_tag=PA14_64950	gene-PA14_64950	pncA		Gene	protein_coding	PA14_64950
**gene-PA14_42600**	CP000438.1	3789550	3790668	+	1119	-2.04702215065279	1.8090618267711	42.2266237514632	0.000110353526005203	0.00617872606283499	BH	group2	glm	ID=gene-PA14_42600;Name=pscP;Note=corresponds to Pseudomonas aeruginosa strain PAO1 locus PA1695;gbkey=Gene;gene=pscP;gene_biotype=protein_coding;locus_tag=PA14_42600	gene-PA14_42600	pscP	corresponds to Pseudomonas aeruginosa strain PAO1 locus PA1695	Gene	protein_coding	PA14_42600
**gene-PA14_34600**	CP000438.1	3073663	3075288	-	1626	-2.04998330901383	7.22613121488253	40.1749862703008	0.00013316434052083	0.00665331081254638	BH	group2	glm	ID=gene-PA14_34600;Name=gapB;gbkey=Gene;gene=gapB;gene_biotype=protein_coding;locus_tag=PA14_34600	gene-PA14_34600	gapB		Gene	protein_coding	PA14_34600
**gene-PA14_06420**	CP000438.1	564699	565457	+	759	-2.05324511574975	3.50577990196106	27.8251546978792	0.000506048692828882	0.0164294059181142	BH	group2	glm	ID=gene-PA14_06420;Name=PA14_06420;Note=corresponds to Pseudomonas aeruginosa strain PAO1 locus PA0492;gbkey=Gene;gene_biotype=protein_coding;locus_tag=PA14_06420	gene-PA14_06420	PA14_06420	corresponds to Pseudomonas aeruginosa strain PAO1 locus PA0492	Gene	protein_coding	PA14_06420
**gene-PA14_18275**	CP000438.1	1567535	1569292	+	1758	-2.11631760820819	4.31520355767045	86.4946735921656	6.40265413807971E-06	0.000839184236688766	BH	group2	glm	ID=gene-PA14_18275;Name=fruA;gbkey=Gene;gene=fruA;gene_biotype=protein_coding;locus_tag=PA14_18275	gene-PA14_18275	fruA		Gene	protein_coding	PA14_18275
**gene-PA14_00560**	CP000438.1	57148	58476	+	1329	-2.12121239525551	5.7182668206551	112.454132215394	2.14483444592405E-06	0.000455566144114397	BH	group2	glm	ID=gene-PA14_00560;Name=exoT;gbkey=Gene;gene=exoT;gene_biotype=protein_coding;locus_tag=PA14_00560	gene-PA14_00560	exoT		Gene	protein_coding	PA14_00560
**gene-PA14_23090**	CP000438.1	2002349	2003011	+	663	-2.12800235774446	5.49876281350174	28.3597077864092	0.000473349338007099	0.015779223308017	BH	group2	glm	ID=gene-PA14_23090;Name=edaA;Note=corresponds to Pseudomonas aeruginosa strain PAO1 locus PA3181;gbkey=Gene;gene=edaA;gene_biotype=protein_coding;locus_tag=PA14_23090	gene-PA14_23090	edaA	corresponds to Pseudomonas aeruginosa strain PAO1 locus PA3181	Gene	protein_coding	PA14_23090
**gene-PA14_50550**	CP000438.1	4490502	4491269	+	768	-2.13428951168392	4.33317556017228	59.125928078798	2.98811828910828E-05	0.00236061344839554	BH	group2	glm	ID=gene-PA14_50550;Name=braF;Note=corresponds to Pseudomonas aeruginosa strain PAO1 locus PA1071;gbkey=Gene;gene=braF;gene_biotype=protein_coding;locus_tag=PA14_50550	gene-PA14_50550	braF	corresponds to Pseudomonas aeruginosa strain PAO1 locus PA1071	Gene	protein_coding	PA14_50550
**gene-PA14_64960**	CP000438.1	5789851	5791050	+	1200	-2.14224623381211	6.25257287634846	61.5092632283762	2.55354874607302E-05	0.00226558701824663	BH	group2	glm	ID=gene-PA14_64960;Name=pncB1;gbkey=Gene;gene=pncB1;gene_biotype=protein_coding;gene_synonym=pncB;locus_tag=PA14_64960	gene-PA14_64960	pncB1		Gene	protein_coding	PA14_64960
**gene-PA14_06480**	CP000438.1	568039	569016	+	978	-2.18337387138255	3.31654854328038	18.8665651033579	0.00185604166686125	0.0440485279538634	BH	group2	glm	ID=gene-PA14_06480;Name=PA14_06480;Note=corresponds to Pseudomonas aeruginosa strain PAO1 locus PA0496;gbkey=Gene;gene_biotype=protein_coding;locus_tag=PA14_06480	gene-PA14_06480	PA14_06480	corresponds to Pseudomonas aeruginosa strain PAO1 locus PA0496	Gene	protein_coding	PA14_06480
**gene-PA14_05770**	CP000438.1	509567	511006	-	1440	-2.18876587252317	3.89352708550272	24.3026437980272	0.000807243626609527	0.0237519081360058	BH	group2	glm	ID=gene-PA14_05770;Name=dhT;gbkey=Gene;gene=dhT;gene_biotype=protein_coding;locus_tag=PA14_05770	gene-PA14_05770	dhT		Gene	protein_coding	PA14_05770
**gene-PA14_42620**	CP000438.1	3791591	3792244	+	654	-2.19477349538059	2.53488647190207	44.9453143310481	8.70263415789411E-05	0.00496911793946291	BH	group2	glm	ID=gene-PA14_42620;Name=pscR;gbkey=Gene;gene=pscR;gene_biotype=protein_coding;gene_synonym=yscR;locus_tag=PA14_42620	gene-PA14_42620	pscR		Gene	protein_coding	PA14_42620
**gene-PA14_42610**	CP000438.1	3790665	3791594	+	930	-2.20799538239605	2.27056569267557	39.5475103846695	0.000141267249984366	0.00690413754796471	BH	group2	glm	ID=gene-PA14_42610;Name=pscQ;gbkey=Gene;gene=pscQ;gene_biotype=protein_coding;gene_synonym=yscQ;locus_tag=PA14_42610	gene-PA14_42610	pscQ		Gene	protein_coding	PA14_42610
**gene-PA14_68080**	CP000438.1	6074299	6074994	+	696	-2.2148420423254	2.73866930136083	59.6970820763989	2.87622543983089E-05	0.00235963215191348	BH	group2	glm	ID=gene-PA14_68080;Name=PA14_68080;Note=corresponds to Pseudomonas aeruginosa strain PAO1 locus PA5154;gbkey=Gene;gene_biotype=protein_coding;locus_tag=PA14_68080	gene-PA14_68080	PA14_68080	corresponds to Pseudomonas aeruginosa strain PAO1 locus PA5154	Gene	protein_coding	PA14_68080
**gene-PA14_34580**	CP000438.1	3072186	3073445	-	1260	-2.214893099286	0.807763404181211	40.8051584144366	0.000125591100700514	0.0066448062177969	BH	group2	glm	ID=gene-PA14_34580;Name=PA14_34580;Note=corresponds to Pseudomonas aeruginosa strain PAO1 locus PA2324;gbkey=Gene;gene_biotype=protein_coding;locus_tag=PA14_34580	gene-PA14_34580	PA14_34580	corresponds to Pseudomonas aeruginosa strain PAO1 locus PA2324	Gene	protein_coding	PA14_34580
**gene-PA14_34640**	CP000438.1	3077071	3077592	-	522	-2.21717401282001	5.26305424882457	85.4593948879362	6.72844617527319E-06	0.000849250431474353	BH	group2	glm	ID=gene-PA14_34640;Name=gntK;gbkey=Gene;gene=gntK;gene_biotype=protein_coding;gene_synonym=gntV,gnuK;locus_tag=PA14_34640	gene-PA14_34640	gntK		Gene	protein_coding	PA14_34640
**gene-PA14_23070**	CP000438.1	2000159	2001628	+	1470	-2.21901765962477	7.11412469959589	34.0075033988752	0.000246876699622208	0.00981888225325016	BH	group2	glm	ID=gene-PA14_23070;Name=zwf;gbkey=Gene;gene=zwf;gene_biotype=protein_coding;locus_tag=PA14_23070	gene-PA14_23070	zwf		Gene	protein_coding	PA14_23070
**gene-PA14_42530**	CP000438.1	3786093	3786464	-	372	-2.24347287953012	-0.119486405562927	29.7585199838471	0.000399255724590074	0.014301290457832	BH	group2	glm	ID=gene-PA14_42530;Name=pcr2;gbkey=Gene;gene=pcr2;gene_biotype=protein_coding;locus_tag=PA14_42530	gene-PA14_42530	pcr2		Gene	protein_coding	PA14_42530
**gene-PA14_50540**	CP000438.1	4489252	4490505	+	1254	-2.24799469229529	4.27527147335861	41.0262983083299	0.00012305894617749	0.00662916568782633	BH	group2	glm	ID=gene-PA14_50540;Name=braE;Note=corresponds to Pseudomonas aeruginosa strain PAO1 locus PA1072;gbkey=Gene;gene=braE;gene_biotype=protein_coding;locus_tag=PA14_50540	gene-PA14_50540	braE	corresponds to Pseudomonas aeruginosa strain PAO1 locus PA1072	Gene	protein_coding	PA14_50540
**gene-PA14_34550**	CP000438.1	3070920	3072155	-	1236	-2.25052453370776	0.0583396644534007	33.9034658661557	0.000249651300423564	0.00986122636673078	BH	group2	glm	ID=gene-PA14_34550;Name=PA14_34550;Note=corresponds to Pseudomonas aeruginosa strain PAO1 locus PA2325;gbkey=Gene;gene_biotype=protein_coding;locus_tag=PA14_34550	gene-PA14_34550	PA14_34550	corresponds to Pseudomonas aeruginosa strain PAO1 locus PA2325	Gene	protein_coding	PA14_34550
**gene-PA14_22890**	CP000438.1	1985515	1986519	-	1005	-2.2749640044965	6.90419941093927	24.0853804969668	0.000832212736028232	0.0242392467104789	BH	group2	glm	ID=gene-PA14_22890;Name=gapA;gbkey=Gene;gene=gapA;gene_biotype=protein_coding;locus_tag=PA14_22890	gene-PA14_22890	gapA		Gene	protein_coding	PA14_22890
**gene-PA14_11810**	CP000438.1	1022458	1023978	-	1521	-2.279667570551	3.30049068621737	114.94295053034	1.95600512229008E-06	0.000433856982317188	BH	group2	glm	ID=gene-PA14_11810;Name=PA14_11810;Note=corresponds to Pseudomonas aeruginosa strain PAO1 locus PA4022;gbkey=Gene;gene_biotype=protein_coding;locus_tag=PA14_11810	gene-PA14_11810	PA14_11810	corresponds to Pseudomonas aeruginosa strain PAO1 locus PA4022	Gene	protein_coding	PA14_11810
**gene-PA14_23080**	CP000438.1	2001615	2002331	+	717	-2.30482916929672	4.74516026393998	37.5168798627247	0.000171968786470225	0.00793395193259031	BH	group2	glm	ID=gene-PA14_23080;Name=pgl;gbkey=Gene;gene=pgl;gene_biotype=protein_coding;locus_tag=PA14_23080	gene-PA14_23080	pgl		Gene	protein_coding	PA14_23080
**gene-PA14_42580**	CP000438.1	3789086	3789562	+	477	-2.31852201950748	0.556756501176161	62.2685834750362	2.43158573239442E-05	0.00219108670604978	BH	group2	glm	ID=gene-PA14_42580;Name=pscO;gbkey=Gene;gene=pscO;gene_biotype=protein_coding;locus_tag=PA14_42580	gene-PA14_42580	pscO		Gene	protein_coding	PA14_42580
**gene-PA14_22990**	CP000438.1	1993669	1994601	+	933	-2.3420547303917	3.19486992745818	69.2332268226644	1.58857579034739E-05	0.00163594938980954	BH	group2	glm	ID=gene-PA14_22990;Name=gltF;Note=corresponds to Pseudomonas aeruginosa strain PAO1 locus PA3189;gbkey=Gene;gene=gltF;gene_biotype=protein_coding;locus_tag=PA14_22990	gene-PA14_22990	gltF	corresponds to Pseudomonas aeruginosa strain PAO1 locus PA3189	Gene	protein_coding	PA14_22990
**gene-PA14_42480**	CP000438.1	3782525	3782821	-	297	-2.35718591883446	0.498008867751816	17.656399065953	0.00228652009397805	0.0499161706245365	BH	group2	glm	ID=gene-PA14_42480;Name=pcrG;gbkey=Gene;gene=pcrG;gene_biotype=protein_coding;locus_tag=PA14_42480	gene-PA14_42480	pcrG		Gene	protein_coding	PA14_42480
**gene-PA14_50530**	CP000438.1	4488332	4489255	+	924	-2.36731488861549	4.38792413926512	36.2202650801301	0.000195887561083575	0.00849386138924044	BH	group2	glm	ID=gene-PA14_50530;Name=braD;Note=corresponds to Pseudomonas aeruginosa strain PAO1 locus PA1073;gbkey=Gene;gene=braD;gene_biotype=protein_coding;locus_tag=PA14_50530	gene-PA14_50530	braD	corresponds to Pseudomonas aeruginosa strain PAO1 locus PA1073	Gene	protein_coding	PA14_50530
**gene-PA14_22980**	CP000438.1	1992306	1993568	+	1263	-2.44682980164385	6.31659039417157	184.251765802986	2.60761830143178E-07	0.000150381347443571	BH	group2	glm	ID=gene-PA14_22980;Name=gltB;Note=corresponds to Pseudomonas aeruginosa strain PAO1 locus PA3190;gbkey=Gene;gene=gltB;gene_biotype=protein_coding;locus_tag=PA14_22980	gene-PA14_22980	gltB	corresponds to Pseudomonas aeruginosa strain PAO1 locus PA3190	Gene	protein_coding	PA14_22980
**gene-PA14_42630**	CP000438.1	3792247	3792513	+	267	-2.4577711226749	0.788650801453274	40.1218866396499	0.000133827649428712	0.00665331081254638	BH	group2	glm	ID=gene-PA14_42630;Name=pscS;gbkey=Gene;gene=pscS;gene_biotype=protein_coding;locus_tag=PA14_42630	gene-PA14_42630	pscS		Gene	protein_coding	PA14_42630
**gene-PA14_42540**	CP000438.1	3786451	3786729	-	279	-2.46027677348905	1.31986111892005	40.5745813004238	0.000128299478324879	0.00664975208444733	BH	group2	glm	ID=gene-PA14_42540;Name=pcr1;gbkey=Gene;gene=pcr1;gene_biotype=protein_coding;locus_tag=PA14_42540	gene-PA14_42540	pcr1		Gene	protein_coding	PA14_42540
**gene-PA14_53070**	CP000438.1	4704698	4705771	-	1074	-2.46290320251621	6.73939069869088	9.69781773267824	0.0123961504765073	0.142407569318761	BH	group2	glm	ID=gene-PA14_53070;Name=hpd;Note=corresponds to Pseudomonas aeruginosa strain PAO1 locus PA0865;gbkey=Gene;gene=hpd;gene_biotype=protein_coding;locus_tag=PA14_53070	gene-PA14_53070	hpd	corresponds to Pseudomonas aeruginosa strain PAO1 locus PA0865	Gene	protein_coding	PA14_53070
**gene-PA14_39690**	CP000438.1	3535976	3538003	+	2028	-2.4730366287256	6.2382935841095	125.09083307946	1.36819922034769E-06	0.000365993306685291	BH	group2	glm	ID=gene-PA14_39690;Name=nrdD;gbkey=Gene;gene=nrdD;gene_biotype=protein_coding;locus_tag=PA14_39690	gene-PA14_39690	nrdD		Gene	protein_coding	PA14_39690
**gene-PA14_42310**	CP000438.1	3771778	3772035	-	258	-2.5058338132816	4.193229069921	143.353724374958	7.66421218042178E-07	0.000232629008655223	BH	group2	glm	ID=gene-PA14_42310;Name=pscF;gbkey=Gene;gene=pscF;gene_biotype=protein_coding;gene_synonym=yscF;locus_tag=PA14_42310	gene-PA14_42310	pscF		Gene	protein_coding	PA14_42310
**gene-PA14_06460**	CP000438.1	567171	568049	+	879	-2.57328428560464	3.90608071101697	41.6347735272989	0.000116406890409174	0.00645498593259331	BH	group2	glm	ID=gene-PA14_06460;Name=PA14_06460;Note=corresponds to Pseudomonas aeruginosa strain PAO1 locus PA0495;gbkey=Gene;gene_biotype=protein_coding;locus_tag=PA14_06460	gene-PA14_06460	PA14_06460	corresponds to Pseudomonas aeruginosa strain PAO1 locus PA0495	Gene	protein_coding	PA14_06460
**gene-PA14_42440**	CP000438.1	3779064	3779951	-	888	-2.62919913153158	4.91129992776181	170.768778518137	3.61903845672655E-07	0.000155545646861789	BH	group2	glm	ID=gene-PA14_42440;Name=popD;gbkey=Gene;gene=popD;gene_biotype=protein_coding;locus_tag=PA14_42440	gene-PA14_42440	popD		Gene	protein_coding	PA14_42440
**gene-PA14_58410**	CP000438.1	5202483	5203937	+	1455	-2.70971277049904	4.9728351418883	147.241504373552	6.83633279608378E-07	0.000219028506861195	BH	group2	glm	ID=gene-PA14_58410;Name=PA14_58410;Note=corresponds to Pseudomonas aeruginosa strain PAO1 locus PA4501;gbkey=Gene;gene_biotype=protein_coding;locus_tag=PA14_58410	gene-PA14_58410	PA14_58410	corresponds to Pseudomonas aeruginosa strain PAO1 locus PA4501	Gene	protein_coding	PA14_58410
**gene-PA14_42460**	CP000438.1	3781116	3781622	-	507	-2.71926073046347	3.39297068263406	153.574132562157	5.70900109970389E-07	0.000193669466717602	BH	group2	glm	ID=gene-PA14_42460;Name=pcrH;gbkey=Gene;gene=pcrH;gene_biotype=protein_coding;locus_tag=PA14_42460	gene-PA14_42460	pcrH		Gene	protein_coding	PA14_42460
**gene-PA14_06450**	CP000438.1	565793	567169	+	1377	-2.79672037355418	4.04785515939499	47.1673681473397	7.23134494419928E-05	0.0044365070524678	BH	group2	glm	ID=gene-PA14_06450;Name=accC;gbkey=Gene;gene=accC;gene_biotype=protein_coding;locus_tag=PA14_06450	gene-PA14_06450	accC		Gene	protein_coding	PA14_06450
**gene-PA14_37250**	CP000438.1	3317016	3318287	+	1272	-2.82136005913356	1.04125500266214	106.533483793903	2.69170928501386E-06	0.000517436248222497	BH	group2	glm	ID=gene-PA14_37250;Name=PA14_37250;Note=corresponds to Pseudomonas aeruginosa strain PAO1 locus PA2114;gbkey=Gene;gene_biotype=protein_coding;locus_tag=PA14_37250	gene-PA14_37250	PA14_37250	corresponds to Pseudomonas aeruginosa strain PAO1 locus PA2114	Gene	protein_coding	PA14_37250
**gene-PA14_42470**	CP000438.1	3781631	3782515	-	885	-2.82504704428416	4.21671212953518	195.113374918593	2.03538579329762E-07	0.000130422998554971	BH	group2	glm	ID=gene-PA14_42470;Name=pcrV;gbkey=Gene;gene=pcrV;gene_biotype=protein_coding;locus_tag=PA14_42470	gene-PA14_42470	pcrV		Gene	protein_coding	PA14_42470
**gene-PA14_42450**	CP000438.1	3779963	3781135	-	1173	-2.89595100566989	5.7888472408157	156.383853041088	5.28217083526083E-07	0.000190389245043432	BH	group2	glm	ID=gene-PA14_42450;Name=popB;gbkey=Gene;gene=popB;gene_biotype=protein_coding;locus_tag=PA14_42450	gene-PA14_42450	popB		Gene	protein_coding	PA14_42450
**gene-PA14_13280**	CP000438.1	1140663	1141886	+	1224	-2.92134608541677	4.76866047215017	8.72485049130717	0.0160758855003218	0.164128751156863	BH	group2	glm	ID=gene-PA14_13280;Name=moeA1;Note=corresponds to Pseudomonas aeruginosa strain PAO1 locus PA3914;gbkey=Gene;gene=moeA1;gene_biotype=protein_coding;locus_tag=PA14_13280	gene-PA14_13280	moeA1	corresponds to Pseudomonas aeruginosa strain PAO1 locus PA3914	Gene	protein_coding	PA14_13280
**gene-PA14_18250**	CP000438.1	1563719	1566589	+	2871	-2.97316426362017	5.66122838560551	196.105747609008	1.99115210111196E-07	0.000130422998554971	BH	group2	glm	ID=gene-PA14_18250;Name=fruB;gbkey=Gene;gene=fruB;gene_biotype=protein_coding;locus_tag=PA14_18250	gene-PA14_18250	fruB		Gene	protein_coding	PA14_18250
**gene-PA14_23000**	CP000438.1	1994594	1995439	+	846	-2.98295501720312	3.22896585758263	169.092518828345	3.77603443049254E-07	0.000155545646861789	BH	group2	glm	ID=gene-PA14_23000;Name=gltG;Note=corresponds to Pseudomonas aeruginosa strain PAO1 locus PA3188;gbkey=Gene;gene=gltG;gene_biotype=protein_coding;locus_tag=PA14_23000	gene-PA14_23000	gltG	corresponds to Pseudomonas aeruginosa strain PAO1 locus PA3188	Gene	protein_coding	PA14_23000
**gene-PA14_35460**	CP000438.1	3152097	3153542	-	1446	-3.01524855228609	3.79531351050809	107.424792696057	2.59931045222596E-06	0.000516904254413348	BH	group2	glm	ID=gene-PA14_35460;Name=PA14_35460;Note=corresponds to Pseudomonas aeruginosa strain PAO1 locus PA2252;gbkey=Gene;gene_biotype=protein_coding;locus_tag=PA14_35460	gene-PA14_35460	PA14_35460	corresponds to Pseudomonas aeruginosa strain PAO1 locus PA2252	Gene	protein_coding	PA14_35460
**gene-PA14_06430**	CP000438.1	565534	565782	+	249	-3.03442471177634	2.65900264278959	37.5521848635994	0.000171369029042279	0.00793395193259031	BH	group2	glm	ID=gene-PA14_06430;Name=PA14_06430;Note=corresponds to Pseudomonas aeruginosa strain PAO1 locus PA0493;gbkey=Gene;gene_biotype=protein_coding;locus_tag=PA14_06430	gene-PA14_06430	PA14_06430	corresponds to Pseudomonas aeruginosa strain PAO1 locus PA0493	Gene	protein_coding	PA14_06430
**gene-PA14_18260**	CP000438.1	1566589	1567533	+	945	-3.13865636522041	4.20927913762182	211.004064582422	1.44933028191982E-07	0.000130422998554971	BH	group2	glm	ID=gene-PA14_18260;Name=fruK;gbkey=Gene;gene=fruK;gene_biotype=protein_coding;locus_tag=PA14_18260	gene-PA14_18260	fruK		Gene	protein_coding	PA14_18260
**gene-PA14_13750**	CP000438.1	1180817	1182112	+	1296	-3.32720502481091	7.97355088807756	9.10152168703887	0.0145097183802492	0.155824107819175	BH	group2	glm	ID=gene-PA14_13750;Name=narK1;gbkey=Gene;gene=narK1;gene_biotype=protein_coding;gene_synonym=narD;locus_tag=PA14_13750	gene-PA14_13750	narK1		Gene	protein_coding	PA14_13750
**gene-PA14_34630**	CP000438.1	3075622	3076974	-	1353	-3.46160082144642	5.44588135024064	269.031011076583	5.02274083221984E-08	0.000130422998554971	BH	group2	glm	ID=gene-PA14_34630;Name=gnuT;gbkey=Gene;gene=gnuT;gene_biotype=protein_coding;gene_synonym=gntP;locus_tag=PA14_34630	gene-PA14_34630	gnuT		Gene	protein_coding	PA14_34630
**gene-PA14_23010**	CP000438.1	1995472	1996632	+	1161	-3.49933368231445	4.49797062407845	169.557600269059	3.7316554999057E-07	0.000155545646861789	BH	group2	glm	ID=gene-PA14_23010;Name=gltK;gbkey=Gene;gene=gltK;gene_biotype=protein_coding;gene_synonym=ugpC;locus_tag=PA14_23010	gene-PA14_23010	gltK		Gene	protein_coding	PA14_23010
**gene-PA14_13260**	CP000438.1	1140109	1140666	+	558	-3.80125938895551	6.48534598257497	16.6889208479496	0.00272134470846091	0.0564532191859499	BH	group2	glm	ID=gene-PA14_13260;Name=moaB1;Note=corresponds to Pseudomonas aeruginosa strain PAO1 locus PA3915;gbkey=Gene;gene=moaB1;gene_biotype=protein_coding;locus_tag=PA14_13260	gene-PA14_13260	moaB1	corresponds to Pseudomonas aeruginosa strain PAO1 locus PA3915	Gene	protein_coding	PA14_13260
**gene-PA14_34610**	CP000438.1	3075453	3075596	-	144	-4.02840029746732	2.05744785918128	160.936064238134	4.67025179983425E-07	0.000179555614197628	BH	group2	glm	ID=gene-PA14_34610;Name=PA14_34610;gbkey=Gene;gene_biotype=protein_coding;locus_tag=PA14_34610	gene-PA14_34610	PA14_34610		Gene	protein_coding	PA14_34610

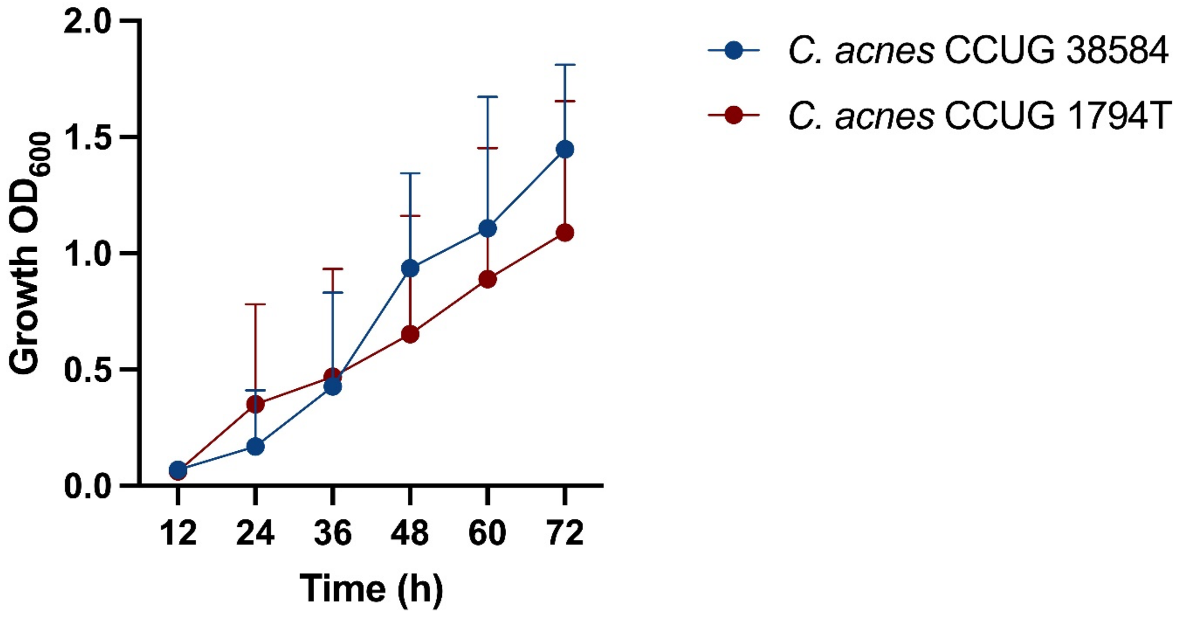

Supplementary Figure 1. The growth curve of *C. acnes* CCUG 38584 and CCUG 1794T measured over 72h. There was no significant difference observed between the two strains. Data includes 3 biological replicates with 3 advanced technical replicates per biological replicate.

Supplementary Figure 2.1. The pathway analysis of the differentially expressed genes. KEGG pathway analysis of *P. aeruginosa* downregulated DEGs in the presence of *C. acnes* CCUG 38584

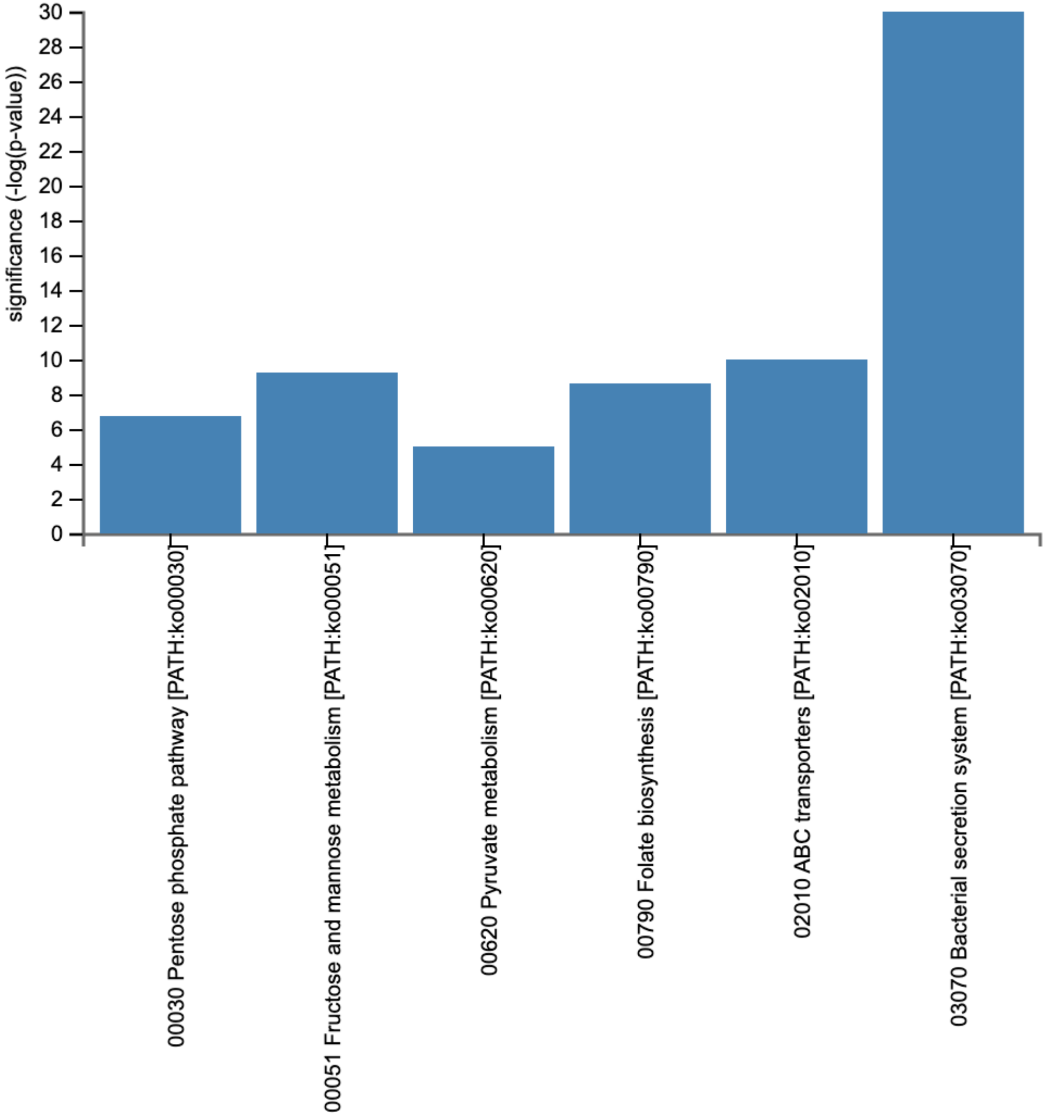

Supplementary Figure 2.2. The pathway analysis of the differentially expressed genes. KEGG pathway analysis of *P. aeruginosa up*regulated DEGs in the presence of *C. acnes* CCUG 38584.

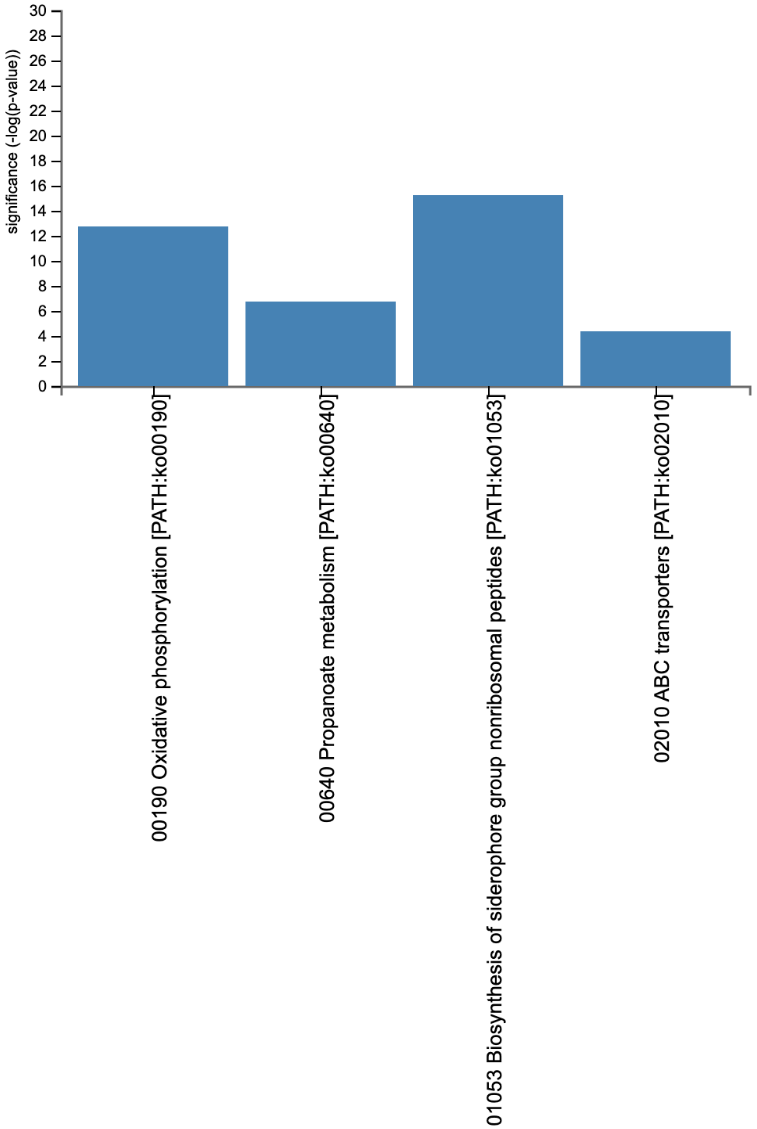

Supplement: Supplementary file 1 — Supplementary Material [file 41522_2024_518_MOESM1_ESM.pdf]
